# Abdominal Pain and Vaginal Discharge: An Eye-Opening Simulation Case about Human Trafficking

**DOI:** 10.21980/J8.52150

**Published:** 2025-10-31

**Authors:** Nicole E Exeni McAmis, Richard S Feinn, Monica R Saxena, Kelly N Roszczynialski

**Affiliations:** * Los Robles Regional Medical Center, Department of Emergency Medicine, Thousand Oaks, CA; ^ UCLA West Valley Medical Center, Department of Emergency Medicine, Los Angeles, CA; † Stanford University, Department of Emergency Medicine, Palo Alto, CA; ** Frank H. Netter MD School of Medicine, Quinnipiac University, North Haven, CT

## Abstract

**Audience:**

The aim of this simulation case is to educate medical students, interns, junior residents, senior residents, nurses, and faculty on how to identify victims of human trafficking in the healthcare setting. This scenario is adaptable for emergency medicine, outpatient clinic settings, and prehospital settings, including EMS personnel as learners.

**Introduction:**

Human trafficking is a profound violation of human rights and a pressing local, national, and global health problem. Victims are reduced to objects for commerce, fueling a $150 billion-dollar industry and representing the second largest source of income for organized crime.^1^,^2^,^3^,^4^ Globally, an estimated 40.3 million people are victims of modern slavery, with more than 70% being women and girls, and one in four victims being children under the age of 18.^3^,^4^ While once perceived as a mostly international problem, prevalence estimates now show 5.4 victims per 1,000 people across the world, with 1.3 victims per 1,000 in the United States for forced labor.^4^

Healthcare providers are among the few professionals likely to encounter victims. Multiple studies show that 28–88% of victims sought medical care while being trafficked.^6^–^9^ These victims are most likely to seek medical care from emergency departments (63.3%), Planned Parenthood clinics (29.6%), private practices (22.5%), urgent care clinics (21.4%), women’s health clinics (19.4%), and neighborhood clinics (19.4%).^8^ Despite this, only a small fraction of emergency physicians report receiving formal training on human trafficking. This highlights the critical need for enhanced education in emergency medicine, where providers are frequently the first point of contact for victims.

**Educational Objectives:**

At the conclusion of this case, learners should be able to: 1) review red flags of identifying victims of human trafficking in healthcare settings, 2) identify common indicators and injuries associated with human trafficking, 3) demonstrate a trauma-informed care approach when interviewing potential victims, 4) list and provide patients with national resources for human trafficking,5) understand federal and state mandatory reporting laws and the role of the healthcare provider, 6) determine best treatment options in patients with limited healthcare access, including counseling on empiric treatment of sexually transmitted infection (STI), 7) review management options for an undesired pregnancy according to local institutional policies and state laws for the senior case.

**Educational Methods:**

This simulation was designed to assess and improve the level of knowledge on identifying victims of human trafficking in the healthcare setting. This session was conducted using standardized patients portraying both the patient and father/trafficker, a faculty member in the nursing role, and a second faculty member in the control booth. The control booth faculty adjusted the displayed vitals, facilitated case progression, and could call in as registration if needed to progress the case. Each case included approximately four to five learners. A pre-brief was provided to the residents prior to the start of the case, explaining the expectations for interacting with standardized patients (SPs) and emphasizing the importance of safety and professionalism. After each scenario concluded, a post-simulation debriefing was held focusing on the presentation, differential diagnosis, physical exam findings, and management of the targeted social and medical issues. This case scenario can also be adapted for use as an oral board examination case.

**Research Methods:**

The authors performed a knowledge assessment of the case using both pre-simulation and post-simulation surveys designed specifically for this project. These surveys measured participants’ knowledge of human trafficking prior to training and their knowledge after the session. Facilitators also provided informal feedback to the scenario developers after the case was piloted. These evaluations were reviewed after implementation. This case was trialed with emergency medicine residents across all training levels (PGY-1 through PGY-4).

**Results:**

Linear mixed models were used to compare pre-session to post-session knowledge of human trafficking, with means reported as descriptive statistics and Cohen’s standardized difference (d) used as a measure of effect size. For ordinal questions, a chi-square test compared pre- and post-session responses. Residents’ post-session perceptions of effectiveness were analyzed using frequency distributions. Statistical analyses were conducted using SPSS v29. Open-ended feedback responses were analyzed qualitatively using content analysis, with each author independently reviewing and categorizing key themes.

Participants reported gaining a deeper understanding of the complexities of human trafficking and greater confidence in their ability to recognize and intervene. A total of 29 residents participated across all four years of training (PGY-1 = 9, PGY-2 = 4, PGY-3 = 11, PGY-4 = 5; 51% female). Only 24% reported prior training, while 94% believed they would benefit from training on human trafficking. Knowledge scores improved significantly (Pre: 59.2 → Post: 65.1; Cohen’s d = 0.39, p < .05). Self-reported comfort recognizing victims increased from 35% to 64% (p < .05), and comfort managing victims increased from 28% to 69% (p < .05), with no differences by PGY level or gender. On the post-survey, 100% of participants agreed the simulation enhanced their knowledge.

Qualitative comments were gathered digitally through a QR code linked to Smartsheet as part of the standard process for resident didactic feedback. Resident responses were provided to case authors without any identifying information, except for PGY year. Prompts for qualitative comments were open-ended response questions of feedback for presenters and their most valuable learning points. Qualitative feedback (n = 27) emphasized increased awareness, the Human Trafficking Hotline as a valuable resource, and strategies for investigating concerns and providing medical management. Many also suggested smaller groups, additional pre-simulation training, and clearer integration of social work. Overall, residents highlighted that this simulation not only improved their base of knowledge but also provided practical tools to support victims in real-world clinical settings.

**Discussion:**

Simulation-based training on human trafficking in emergency medicine is a vital tool for preparing providers to recognize and respond to these complex cases. By engaging in highly interactive, standardized patient scenarios, learners can practice recognizing subtle red flags, applying trauma-informed communication, and balancing confidentiality with mandated reporting requirements. The debriefing sessions allow further reflection, knowledge integration, and discussion of best practices. Although standardized patients may be cost-prohibitive, faculty can serve as role players to reduce barriers to implementation. Through such training, healthcare providers enhance preparedness, empathy, and effectiveness in addressing the needs of trafficking survivors and contribute to broader efforts to combat exploitation.

**Topics:**

Medical simulation, emergency medicine, human trafficking, sex trafficking, sexually transmitted diseases, abuse, non-accidental trauma, domestic abuse.

## USER GUIDE

**Table:** 

List of Resources:	
Abstract	1
User Guide	3
Instructor Materials	8
Standardized Patient Briefing Materials	21
Operator Materials	30
Debriefing and Evaluation Pearls	34
Simulation Assessment	37

**Learner Audience:**

Medial Students, Interns, Junior Residents, Senior Residents, With modifications this case could be set in the pre-hospital or outpatient clinic setting to expand learner population

**Time Required for Implementation:**

**Instructor Preparation: Total 3 hours:** 1 hour for self-study of the facilitator, 2 hours for training of the Standardized Patients (SP) or faculty members serving as the patient and trafficker for the case. A trial run with the SP team may be helpful to ensure consistency and familiarity with the case. Please refer to the separate SP Scripting Guide for training the SPs for this case

**Time for case:** 15 min

**Time for debriefing:** 25 min

**Recommended Number of Learners per Instructor:**

1–5

**Topics:**

Medical simulation, emergency medicine, human trafficking, sex trafficking, sexually transmitted diseases, abuse, non-accidental trauma, domestic abuse.

**Objectives:**

By the end of the session:

1. Review red flags of identifying victims of human trafficking in healthcare settings
2. Identify common indicators and injuries associated with human trafficking
3. Demonstrate a trauma-informed care approach when interviewing potential victims
4. List and provide patients with national resources for human trafficking
5. Understand federal and state mandatory reporting laws and the role of the healthcare provider
6. Determine best treatment options in patients with limited healthcare access, including counseling on empiric treatment of sexually transmitted infection (STI)
7. (For Senior Case) Review management options for an undesired pregnancy according to local institutional policies and state laws

### Linked objectives and methods

We applied Kolb’s Experiential Learning Cycle to structure this simulation.^10^

The concrete experience occurs during the 15-minute simulation, with reflection and abstract conceptualization occurring in the debrief, and active experimentation applied to future clinical practice. Each learning objective is explicitly addressed through the simulation design, debriefing, and supplemental teaching as follows:

1. Review the red flags of human trafficking in healthcare settings.
  During the simulation, learners observe concerning dynamics such as the patient’s withdrawn behavior and the father/trafficker’s controlling presence. The embedded nurse and faculty prompts reinforce these cues. In debriefing, facilitators highlight how these behaviors represent red flags.^1^,^7^
2. Identify common indicators and injuries associated with trafficking.
  The physical exam stimuli (bruising on the thighs, track marks, cervical discharge) are designed to mirror common trafficking-related findings. In the debrief, faculty review how these medical and social clues integrate into a broader suspicion of trafficking.
3. Demonstrate a trauma-informed care approach when interviewing potential victims.
  Learners must attempt to separate the patient from the trafficker, establish rapport, and conduct a sensitive history. The debrief reinforces trauma-informed strategies such as sitting at eye level, normalizing silence, and respecting patient autonomy.
4. List and provide patients with national resources for victims of trafficking.
  The simulation provides opportunities for learners to share the National Human Trafficking Hotline and other resources once disclosure occurs. Facilitators review different delivery methods (shoe card, memorization, text “BeFree”) in the debrief.
5. Understand federal and state mandatory reporting laws and the provider’s role.
  During the debrief, faculty lead a discussion of reporting requirements, emphasizing differences between minors (mandatory reporting) and adults (report only with consent in most states).
  Supplemental teaching includes review of local/state policies.
6. Determine best treatment options for patients with limited healthcare access, including STI empiric treatment.
  In the simulation, learners diagnose cervicitis and must counsel empiric antibiotic therapy given the patient’s limited follow-up access. The debrief reinforces CDC guidelines and emphasizes adapting care when patients may not reliably return.
7. (Senior case) Review management options for an undesired pregnancy according to local institutional policies and state laws.
  Senior residents encounter a positive pregnancy test and must explore whether the pregnancy is desired. Faculty guide a discussion on counseling, management options (including medical abortion if appropriate), and the importance of following institutional protocols and state regulations.

By mapping each objective to a specific element of the simulation, debrief, or supplemental lecture, this case ensures that learners not only experience the scenario but also reflect, consolidate knowledge, and plan for application in future patient care. Each objective was written according to Bloom’s Revised Taxonomy.^11^

### Recommended pre-reading for instructor

- Shandro J, Chisolm-Straker M, Duber HC, et al. Human trafficking: A guide to identification and approach for the emergency physician. *Ann Emerg Med*. 2016;68(4):501–508.e1. doi:10.1016/j.annemergmed.2016.03.049
- Exeni McAmis, Nicole E. CalACEP Human Trafficking Guide. California ACEP. californiaacep.org/page/HumanTrafficking.
- Exeni McAmis NE, Mirabella AC, McCarthy EM, et al. Assessing healthcare provider knowledge of human trafficking. PLOS ONE. 2022;17(3): e0264338. https://doi.org/10.1371/journal.pone.0264338

### Results and tips for successful implementation

We implemented two versions of this simulation—one for junior residents (PGY-1/2) and one for senior residents (PGY-3/4)—with progressive clinical complexity. The first iteration, intended for junior learners (PGY-1 and PGY-2 emergency medicine residents), did not include the complicating factor of an undesired pregnancy. This factor was introduced only for senior residents (PGY-3 and PGY-4). Junior and senior learner groups were held on separate days, with learners of different years within each designation participating together.

Simultaneous one-hour sessions were conducted, with a total of four simulation cases each day. Junior sessions were held one week, followed by senior sessions the next week during the standing emergency medicine residency conference. Each simulation included three to five learners and two standardized patients (the patient and her father/trafficker). The same standardized patients participated in all sessions, and the patient–father pairings were maintained for consistency.

A simulated nurse role was played by a faculty member in each room for each case. Each session had a total of four faculty members, with two faculty assigned per case: one as the embedded nurse in the room and the other in the control booth, responsible for adjusting vital signs and facilitating the scenario. The second faculty member could also assume an optional role during registration, signaling the team if an abnormal experience occurred; however, this was not required in any case iteration.

Across both sessions, a total of 29 residents participated (PGY-1=9, PGY-2=4, PGY-3=11, PGY-4=5). On the pre-survey, only 24% reported prior training on human trafficking, though 94% believed they would benefit. Knowledge scores improved significantly following the session (Pre: 59.2 → Post: 65.1; Cohen’s d = 0.39, p < .05). Comfort recognizing victims increased from 35% to 64% (p < .05), and comfort managing victims increased from 28% to 69% (p < .05). No differences were observed by PGY level or gender, suggesting the simulation was equally effective across training stages. Post-survey, 100% of residents agreed or strongly agreed that the simulation enhanced their knowledge.

Anonymous written feedback using the standard qualitative conference feedback form was obtained from 27 participants. On this form, the learners were asked two questions: “What did you learn from this session?” and “What feedback do you have for the presenter(s)?” Of these, 63% emphasized that the case significantly enhanced their knowledge and awareness of human trafficking, and many expressed gratitude for the opportunity to engage with such a challenging but relevant scenario. The remaining 37% offered constructive feedback for future improvements, specifically recommending smaller groups (two-three learners per case) to allow for more hands- on participation, additional just-in-time education prior to the simulation, and clearer integration of other interdisciplinary roles, particularly social work. The **learning points** collected from participants further reinforced the value of the simulation. Residents consistently highlighted the Human Trafficking Hotline as a critical resource, noting its usefulness not only for identifying victims but also for providing safe referral options and guidance on management. Others identified skills such as tactfully separating patients from controlling companions, employing trauma-informed communication strategies, and balancing medical care with complex social needs. Compiled feedback and learning points are included below to illustrate how the case translated into actionable skills and knowledge:

**Table:** 

PGY	FEEDBACK
PGY1	This sim should maybe be max 2 providers, 3rd person doesn’t have a ton to do
PGY1	Grateful for this experience and the helpful debrief - thank you!
PGY1	Kind of a hard one to do with multiple people at a time
PGY1	It would be great if we had training before the sim so we could use it as a chance to practice skills
PGY1	Great job!!! Super hands on thanks for doing this important work.
PGY1	This was a great case!
PGY1	It was so powerful and I’m so glad that I was able to participate. Thank you!
PGY1	Would have appreciated just-in-time learning before to better know some useful tools to incorporate. Otherwise good session.
PGY1	Thank you for the survey!
PGY2	This was a fantastic sim and I really enjoyed using a standardized patient because it allowed for a lot more emotion and created a true sense of reality in the case.
PGY2	Thought this was very helpful. Would have appreciated a more institution specific approach. Specifically what do our SW [social workers] do
PGY2	It was excellent, the actors did a great job
PGY2	great sim of a social EM case notwithstanding what medical concerns to be mindful of
PGY3	Good case. Appreciate tips from discussion.
PGY3	Liked the use of real people rather than mannequins for this sim
PGY3	Good case. Hand out would be helpful.
PGY3	How to deal with difficult trafficking cases
PGY3	Great sim for a case we don’t typically encounter
PGY3	Super interesting and challenging sim
PGY3	This was one that needed more time than what’s typically allotted for sim
PGY3	Great case. Thank you!
PGY3	Great sim thank you
PGY3	appreciate these high yield
PGY4	Great case. Very hard.
PGY4	Good case.
PGY4	very valuable sim, thank you for all the effort into setting it up!
PGY4	Great case!

**Table:** 

PGY	LEARNING POINTS
PGY1	Trafficking help line = BeFree
PGY1	How to identify and best serve patients who are victims of human trafficking
PGY1	Think about med management not just the social situation and also remember the hotline
PGY1	Signs of human trafficking and ways to approach the conversation
PGY1	Consider human trafficking by having a low threshold to ask screening questions, involve SW [social workers] and provide resources as needed.
PGY1	I learned about key features to look for in human trafficking.
PGY1	I learned about resources that I can utilize for patients who are human trafficking victims
PGY1	Methods to identify human trafficking and how to negotiate patient care with a victim and other people in the room.
PGY1	The survey prior to sim was a good primer.
PGY2	Learned about identifying victims of human trafficking. Also learned strategies to allow for examining these patients alone and what resources are available.
PGY2	Human trafficking care
PGY2	Signs of trafficking and next steps to take
PGY2	signs of human trafficking how to manage/ navigate /support a patient experiencing this
PGY3	Standard procedure to ask patient guest to leave room for exam. Give patient the trafficking hotline number.
PGY3	Learned how to manage both medical and social aspects of a trafficking case
PGY3	How to identify human trafficking
PGY3	Human trafficking
PGY3	Trauma informed care and questions
PGY3	learned about human trafficking resources
PGY3	Human trafficking patient encounter, how to manage.
PGY3	Find a tactful way to get a patient alone when concerned.
PGY3	Human trafficking
PGY3	Thanks for the faculty facilitators
PGY4	Human trafficking is common and complicated
PGY4	Learned family members are the most likely to traffic
PGY4	doctors can also call the human trafficking help line for advice
PGY4	Human trafficking resources

From an implementation standpoint, several key lessons emerged. Consistency among standardized patients (SPs) was crucial for fidelity across sessions, and the presence of an embedded faculty nurse ensured smooth case flow and timely prompts. Between the junior and senior iterations, one small but impactful modification was made—removing sunglasses from the patient role—which reduced overt signaling and forced learners to rely on more nuanced clinical and behavioral cues. Faculty also noted that the structured debrief was essential because many learners reported that the debrief was the moment when they consolidated knowledge, integrated resources, and gained confidence in applying what they learned.

Finally, given the sensitive nature of the case, attention to SP wellbeing proved critical. SPs portraying the trafficker role described the experience as particularly emotionally challenging, and providing time and space for shedding the role was vital to ensuring their psychological safety. This aligns with the Association of Standardized Patient Educators’ (ASPE) safe work environment guidelines and should be considered essential for future implementation.^12^

Based on resident feedback, this case would have been more appreciated in even smaller groups of two to three residents if possible, allowing each participant to actively engage and practice communication skills, trauma-informed care, and management of the patient’s complex medical needs.

## Supplementary Information



## Figures and Tables

**Table:** 

#1	Left Antecubital Fossa
#2	Left Medial Leg
#3	Point of Care Glucose
#4	CBC
#5	CMP
#6	Electrolytes: Calcium/Magnesium/Phosphorus
#7	UA
#8	VDRL/RPR for syphilis
#9	Rapid HIV
#10	Junior Case B-HCG for pregnancy
#11	Senior Case B-HCG
#12	Senior Case US Pelvis
#13	Senior Case Type and Screen

**Table:** 

**Vitals:**	Heart Rate (HR) 109	Blood Pressure (BP) 124/68
	Respiratory Rate (RR) 20	Temperature (T) 9.9°F
	Oxygen Saturation (O_2_Sat) 98% on room air	

**Figure:**
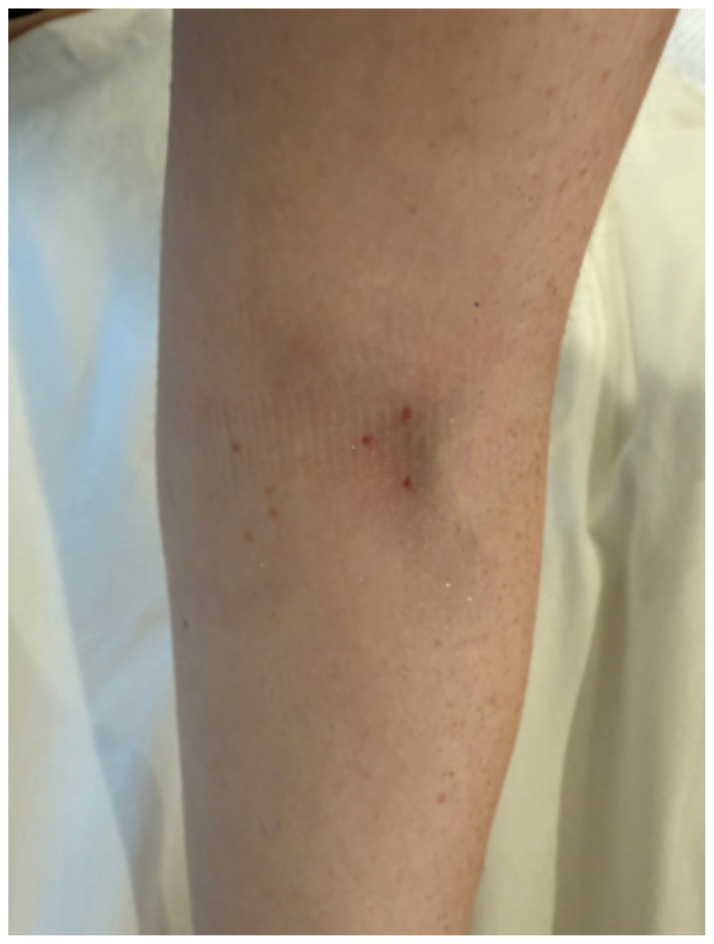


**Figure:**
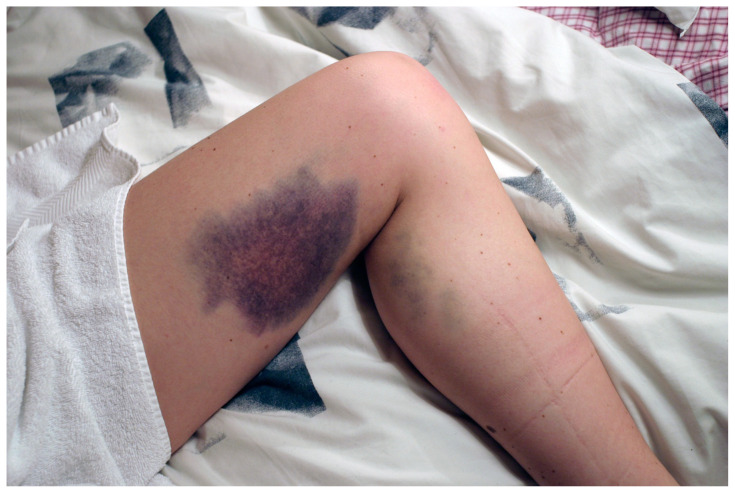


**Table:** 

*Point of Care Glucose*	90 mg/dL

**Table:** 

*CBC (Complete Blood Count)*	
White blood count (WBC)	7.2 × 1000/mm^3^
Hemoglobin (Hgb)	10.9 g/dL
Hematocrit (HCT)	35%
Platelet (Plt)	176 × 1000/mm^3^

**Table:** 

*Basic metabolic panel (BMP)*	
Sodium	135 mEq/L
Potassium	3.7 mEq/L
Chloride	101 mEq/L
Bicarbonate (HCO3)	24 mEq/L
Blood Urea Nitrogen (BUN)	16 mg/dL
Creatinine (Cr)	0.75 mg/dL
Glucose	82 mg/dL

**Table:** 

*Electrolytes*	
Ca	8.9 mg/dL
Mg	2.9 mg/dL
Phos	3.2 mg/dL

**Table:** 

*Urinalysis (UA)*	
pH	6
Specific gravity	1.015
Glucose	0 mg/dL
Protein	20 mg/dL
Urobilinogen	0 mg/dL
Blood	0 mg/dL
Ketone	0 mg/dL
Nitrite	Negative
Leukocytes	25 WBC/uL

**Table:** 

*VDRL/RPR*	
VDRL/RPR	Negative

**Table:** 

*Rapid HIV*	
Rapid HIV	Negative

**Table:** 

*Junior Case B-Hcg*	
B-Hcg	<1 Negative

**Table:** 

*Senior Case B-Hcg*	
B-Hcg	38,000

**Figure:**
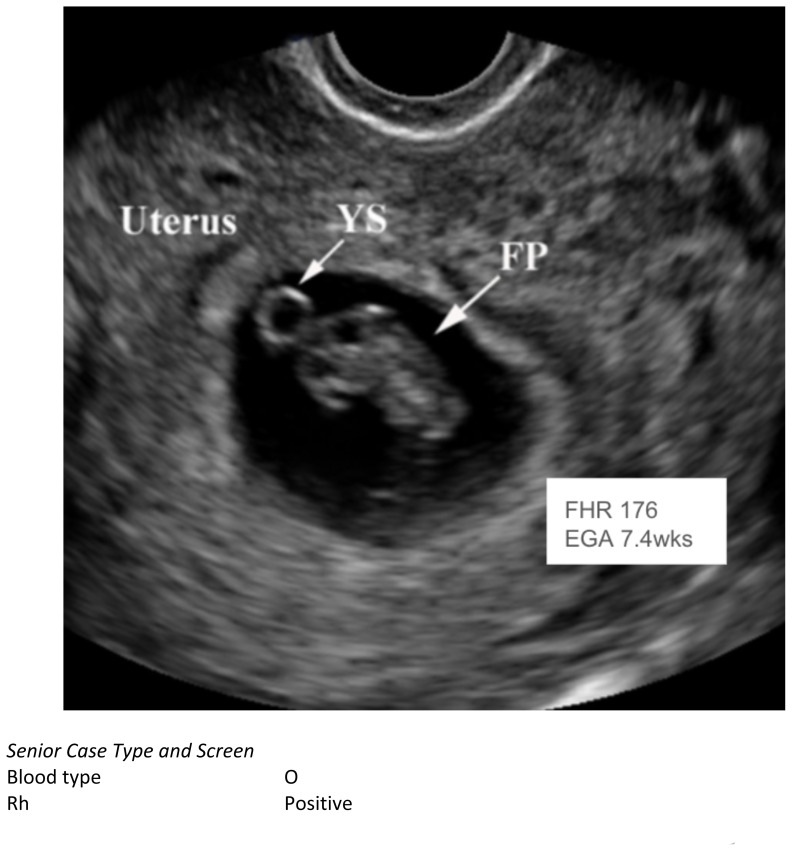


**Table:** 

*Senior Case Type and Screen*	
Blood type	O
Rh	Positive

**Table:** SIMULATION EVENTS TABLE:

Minute (state)	Participant action/ trigger	Patient status (simulator response) & operator prompts	Monitor display (vital signs)
0:00 (Baseline)	Initial assessment and separation of father/trafficker.	**Patient vocalizations:** Father gives history, patient does not vocalize much. Appears anxious, quiet and withdrawn, looking constantly to father. **Expected learner actions:** Get patient on monitor, ask nurse for IV. Separate the father from the patient. Father/trafficker will exit the room after three requests and explanation of need for sensitive exam. Team calmly escorts him out of the room for the sensitive exam. **Operator notes/prompts:** If team does not communicate need for privacy to perform a sensitive pelvic exam, the embedded nurse will cue the team to use this strategy. **Transition to next state:** After father leaves room.	T 98.8º F HR 109 BP 124/68 RR 20 O2 98%
3:00	Interview.	**Patient vocalizations:** Still hesitant but once team gives safe space and support, sits down eye to eye, explains their concern of and interest in purely providing help. Patient shares symptom of vaginal discharge and answers questions regarding sexual and OB history. Does not yet discuss her job or trafficking unless specifically asked. **Expected learner actions:** Sit to meet eye to eye, explain the line of questioning, reassure that patient has control over each next step while in the room, on exam, meds, decisions. Order labs, UA, STI screening tests. Order antiemetic. **Operator notes/prompts:** Nurse can prompt for antiemetics if team does not based on reported symptoms. **Transition to next state:** After meds, and diagnostics return.	T 98.8º F HR 109 BP 124/68 RR 20 O2 98%
5:00	Exam.	**Patient Vocalizations:** Patient states she bruises easily if her lower extremities are examined. Patient shares with the team her IV heroin use if asked about her forearm scars. Upon the nurse sharing the pelvic exam findings, the patient becomes upset and may cry, asking “Do I have a disease? That will affect my life and job.” **Expected Learner Actions:** Reassure the patient and counsel her on treatments for STIs. Inquire what the patient means about affecting her job and life to investigate for domestic violence and trafficking. **Operator notes/prompts:** If residents need prompting to investigate social history further, the faculty in the control room can call in as “registration” (played by the faculty member in the control room), who calls the team to apologize for causing patient distress when asking about insurance and identification. **Transition to next state:** When learners inquire specifically about the patient’s job, and investigate for domestic violence or trafficking.	T 98.8º F HR 79 BP 124/68 RR 20 O2 98%
8:00	Identify Human Sex Trafficking.	**Patient vocalizations:** Patient agrees to medications for STI without confirmatory testing, and after being asked specifically about her job, discloses she is being trafficked. **Expected learner actions:** Counsel on concern based on risk factors and exam for STI and recommend treatment. Offer support and assistance with a consult to social work, law enforcement, or national human trafficking hotline. **Transition to next state:** After disclosure of trafficking.	T 98.8º F HR 79 BP 124/68 RR 20 O2 98%
11:00	Senior Case: Counseling on pregnancy.	**Patient vocalizations:** Upset when informed of positive pregnancy test, if not yet disclosed, can prompt team to ask about trafficking/job by saying “I can’t be pregnant! I can’t bring another child into my life!” Does not want to proceed with the pregnancy and requests termination options. Asks if the follow up appointments can be for the infection and that the team not disclose the pregnancy to father. **Expected learner actions:** Inform patient of positive pregnancy result; if not yet ordered send type and screen to determine Rh status and US to confirm intrauterine pregnancy (IUP). Inquire whether pregnancy is desired and counsel on options for management. Follow institutional and state guidelines and offer OB services in the ED vs outpatient clinic. **Operator notes/prompts:** B-HCG returns positive and US returns with IUP 7.4 weeks. **Transition to next state:** After a plan is established for pregnancy management and follow-up care.	
11:00	Counseling for safety and discharge.	**Patient vocalizations:** The patient does not want law enforcement or social work involved. Is willing to take national human trafficking hotline number if she could hide it from father. Feels better after the medications and can tolerate PO and her first dose of antibiotics. Ultimately decides to be discharged home. **Expected learner actions:** Express support, encouragement to return or reach out for any further concerns, provide trafficking number written down to place in shoe so father won’t see. Provide STI empiric treatment. **Operator notes/prompts:** End of case.	T 98.8º F HR 79 BP 124/68 RR 20 O2 98%

**Table:** 

**Critical Actions:** Identify the father’s overbearing personality and request a private interview with the patient. Obtain focused history and physical exam with father outside the room, including sexual and obstetric history. Obtain appropriate labs, including STI testing and imaging for abdominal pain evaluation. Provide appropriate empiric treatment for STIs and ensure follow-up. Maintain a safe, confidential environment for patient disclosure.	0:00

**Table:** 

	Milestone	Did not achieve level 1	Level 1	Level 2	Level 3
1	**Emergency Stabilization (PC1)**	□ Did not achieve Level 1	□ Recognizes abnormal vital signs	□ Recognizes an unstable patient, requiring intervention Performs primary assessment Discerns data to formulate a diagnostic impression/plan	□ Manages and prioritizes critical actions in a critically ill patient Reassesses after implementing a stabilizing intervention
2	**Performance of focused history and physical (PC2)**	□ Did not achieve Level 1	□ Performs a reliable, comprehensive history and physical exam	□ Performs and communicates a focused history and physical exam based on chief complaint and urgent issues	□ Prioritizes essential components of history and physical exam given dynamic circumstances
3	**Diagnostic studies (PC3)**	□ Did not achieve Level 1	□ Determines the necessity of diagnostic studies	□ Orders appropriate diagnostic studies. Performs appropriate bedside diagnostic studies/procedures	□ Prioritizes essential testing Interprets results of diagnostic studies Reviews risks, benefits, contraindications, and alternatives to a diagnostic study or procedure
4	**Diagnosis (PC4)**	□ Did not achieve Level 1	□ Considers a list of potential diagnoses	□ Considers an appropriate list of potential diagnosis May or may not make correct diagnosis	□ Makes the appropriate diagnosis Considers other potential diagnoses, avoiding premature closure
5	**Pharmacotherapy (PC5)**	□ Did not achieve Level 1	□ Asks patient for drug allergies	□ Selects an medication for therapeutic intervention, consider potential adverse effects	□ Selects the most appropriate medication and understands mechanism of action, effect, and potential side effects Considers and recognizes drug-drug interactions
6	**Observation and reassessment (PC6)**	□ Did not achieve Level 1	□ Reevaluates patient at least one time during case	□ Reevaluates patient after most therapeutic interventions	□ Consistently evaluates the effectiveness of therapies at appropriate intervals
7	**Disposition (PC7)**	□ Did not achieve Level 1	□ Appropriately selects whether to admit or discharge the patient	□ Appropriately selects whether to admit or discharge Involves the expertise of some of the appropriate specialists	□ Educates the patient appropriately about their disposition Assigns patient to an appropriate level of care (ICU/Tele/Floor) Involves expertise of all appropriate specialists
9	**General Approach to Procedures (PC9)**	□ Did not achieve Level 1	□ Identifies pertinent anatomy and physiology for a procedure Uses appropriate Universal Precautions	□ Obtains informed consent Knows indications, contraindications, anatomic landmarks, equipment, anesthetic and procedural technique, and potential complications for common ED procedures	□ Determines a back-up strategy if initial attempts are unsuccessful Correctly interprets results of diagnostic procedure
20	**Professional Values (PROF1)**	□ Did not achieve Level 1	□ Demonstrates caring, honest behavior	□ Exhibits compassion, respect, sensitivity and responsiveness	□ Develops alternative care plans when patients’ personal beliefs and decisions preclude standard care
22	**Patient centered communication (ICS1)**	□ Did not achieve level 1	□ Establishes rapport and demonstrates empathy to patient (and family) Listens effectively	□ Elicits patient’s reason for seeking health care	□ Manages patient expectations in a manner that minimizes potential for stress, conflict, and misunderstanding. Effectively communicates with vulnerable populations, (at risk patients and families)
23	**Team management (ICS2)**	□ Did not achieve level 1	□ Recognizes other members of the patient care team during case (nurse, techs)	□ Communicates pertinent information to other healthcare colleagues	□ Communicates a clear, succinct, and appropriate handoff with specialists and other colleagues Communicates effectively with ancillary staff

## INSTRUCTOR MATERIALS

**Case Title:** Human Trafficking presenting as Abdominal Pain

**Case Description & Diagnosis (short synopsis):** A 26-year-old female presents to the ED for generalized abdominal pain for five days. The patient is accompanied by an older man who identifies as her father. The patient is withdrawn in his presence, allowing him to dominate the conversation with the care team. The team must separate the patient from the father to conduct a full interview and evaluation. The father initially refuses to leave for further questioning until the team explains the need for a sensitive pelvic exam and that standard protocol requires only a medical chaperone if the patient is over age 18.

Once separated, a detailed history and examination reveal four previous pregnancies (one living child), three prior abortions, intravenous heroin use, and multiple sexual partners, both male and female. The team should counsel the patient on STI testing and empiric treatment based on cervical discharge found on patient’s exam, reported by the embedded simulated nurse. If learners struggle to elicit the social history, the control room faculty can call in as “registration” to provide additional cues (eg, patient lacking ID and appearing distressed).

For the junior residents, blood work is reassuring and the pregnancy test is negative. For senior residents, the patient has a positive pregnancy test and confirmed intrauterine pregnancy on ultrasound. Learners should confirm she is Rh positive and counsel the patient on pregnancy management options in accordance with institutional polices and state regulations when the patient reports this is an undesired pregnancy.

There are multiple cues suggesting the patient is a victim of sex trafficking. The most prominent occurs when the patient reacts strongly to discussion of pelvic exam finding and treatment options, stating these would interfere with her “job.” A secondary cue may come via the “registration” call, reinforcing the concern for trafficking. Learners are expected to share resources such as the National Human Trafficking Hotline and social work support. While the patient declines immediate intervention, the team should provide resources discreetly and create a safe space for return. The case concludes as the patient agrees to empiric STI treatment, thanks the team for their care, and requests discharge. The senior case should also include a referral to obstetrics for further management of the undesired pregnancy.

**Equipment or Props Needed:**

- bruising over left inner thighs
- injection site scars to bilateral ACs (antecubital fossae)
- either moulage on the SP with dark eyeshadow and a red pen or displayed on slides)

**Actors needed:**

- PATIENT - Female in her 20s-30s, wearing street clothes. Withdrawn when the father/trafficker is present.
- FATHER/TRAFFICKER – Male in his 40s-50s. Will need to be in the room with the patient at the start of the case. Provides all medical history, interrupts frequently, and resists leaving the room. Will only leave after repeated requests or explanation of exam protocol.
- NURSE - Faculty member who assists with IV placement, draws labs, escorts the father from the room, and keeps him outside as needed.
- (Optional) REGISTRATION – Played by control room faculty, calling in to note that patient lacked documentation (driver’s license, ID, or passport) and appeared anxious.

**Stimulus Inventory:**

**Pre-Brief Scripting to include:** Today, you will be working with standardized patients (SPs). Do not perform any invasive procedures or sensitive exams. Always ask before adjusting or removing clothing, and do not proceed if the SP declines. Otherwise, treat the SP as you would a real patient. If you are unable to continue due to physical, mental, or emotional concerns, use the safety phrase “this is not a simulation.” This safety phrase can be used by faculty or SPs if needed.

**Background and brief information:** A pre-brief introduces the SP simulation, outlines expectations, and reviews the safety phrase. The setting is an academic tertiary care emergency department. The patient arrives by private vehicle with her father and is waiting in the exam room when the resident team enters after being notified by the embedded nurse that there is a new patient to evaluate.

**Initial presentation:** The patient is awake and alert but avoids eye contact, focused on her father. She is in plain clothes on the gurney and is accompanied by her father, who is sitting in a chair beside her. He dominates history-taking, even when questions are directed to the patient. In the junior case, she wears sunglasses and short sleeves with her forearms exposed (track marks visible). For the senior case, the patient does not wear sunglasses and wears long sleeves (track marks covered). She looks to her father for approval before responding. The father reports the initial history, including five days of abdominal pain, nausea, and vomiting, with no fever, chills, chest pain, shortness of breath, diarrhea, or vaginal bleeding. This controlling behavior should prompt learners to separate the father for further evaluation.

**How the scenario unfolds:** The patient presents as shy and reluctant to speak in the father’s presence. The father dominates the conversation, providing most of the history and frequently interrupting the patient. His hovering, defensive behavior raises concern, prompting the team to request a private interview with the patient. Both the patient and father initially resist when asked to step outside.

The father will only leave after being asked three separate times and after the physician explains that a sensitive exam for a patient over 18 is standard procedure with a medical not family chaperone.

A thorough history may reveal four previous pregnancies (one living child), three prior abortions, heroin use via injection, and multiple sexual partners of any gender. The patient initially withholds details about her job but may disclose a history of sex trafficking if trust is established.

If the team struggles to elicit a social history or recognize potential domestic violence or trafficking, a faculty member in the control room can intervene as “registration” to apologize for causing distress due to lack of identification and insurance, signaling to learners the importance of trauma-informed care.

FATHER REMAINS OUT OF THE ROOM FOR THE EXAM.

On exam, the patient has minimal suprapubic abdominal tenderness but tolerates palpation. The team, if they examine her extremities, will note venipuncture scars on bilateral forearms and bruising on her medial thighs (these can be done with moulage or shown in PowerPoint). The plan to perform a pelvic exam is voiced by learners, but is not to be performed on the SP. The embedded nurse reports normal anatomic structures with cervical discharge, closed os, and no cervical motion tenderness.

The team should request labs to be drawn, including a CBC, BMP, Ca/Mg/Phos, B-Hcg, STD panel including HIV and syphilis testing, Type & Rh, and a Urinalysis. The team provides ondansetron for her nausea and inability to tolerate PO prior to arrival. After labs and imaging, she can tolerate a PO challenge.

The team may request a pelvic ultrasound. For the junior case, the ultrasound is negative for intra-uterine pregnancy, ovarian torsion, or tubo-ovarian abscess (faculty or embedded nurse provides the reading).

Upon the discovery of the cervical discharge on the pelvic exam, the team should discuss the need for testing and consideration of empiric treatment for sexually transmitted infections with the patient. The patient gets upset, even crying upon hearing this, asking the team, “Do I have a disease? That would impact my life and my job.” Residents should provide reassurance, emphasizing confidentiality, which can help the patient disclose sensitive social history. If the patient has not yet disclosed her job as a sex worker, the team should ask additional questions regarding her job and what she means by the above statement.

Once trust and confidentiality are established, she provides additional history. Patient discloses that she is a sex worker coerced by her father, who arranges clients and controls her earnings. She earns approximately $2,000 per night to see her five-year-old daughter, with whom her contact is contingent upon meeting this quota. She typically engages with 8–10 clients per night, often unprotected. The patient discloses ongoing heroin use, provided by her father, though she has abstained from use for the past five days due to illness. She describes using heroin to cope with the trauma of her experiences and expresses a desire to quit but feels unable to do so due to availability and lack of support. The patient reports feeling unsafe at home and expresses fear for her and her daughter’s safety. She denies current suicidal or homicidal ideation but reports persistent depression and anxiety related to her living situation and ability to see her daughter.

The team should provide supportive, trauma-informed care, ensuring the patient feels safe and respected. They should discuss empiric treatment for STIs, reassuring the patient that nothing she shares will be disclosed outside the team. Rapid HIV and VRDL/RPR are negative, and the patient agrees to treatment with ceftriaxone, doxycycline, and metronidazole while awaiting the remaining labs.

The team should review available resources for victims of human trafficking, including referrals to social work, the national human trafficking hotline (NHTH), or local county law enforcement agency. While the patient will ultimately decline these resources, residents can offer strategies for safe access, such as a discreet shoe card, memorizing contact numbers, or using text resources like BEFREE, empowering the patient to seek help if needed.

**Senior Case Adjustment:** For senior residents, the patient presents with long sleeves covering her arms, requiring the team to ask her to roll them up to identify venipuncture scars and bruising. A positive pregnancy test is revealed and type and screen show O positive. Ultrasound confirms a viable intrauterine pregnancy at approximately 7.4 weeks gestation (gestational sac, fetal pole, B-HCG 38,000). The patient expresses concern due to her inability to continue working while pregnant and indicates that the pregnancy is undesired.

The senior residents should discuss management options, including medical abortion per institutional protocol if the patient is less than 10 weeks by transvaginal ultrasound. This includes consulting Obstetrics to order mifepristone in the ED and providing a prescription for misoprostol to be taken at home 48–72 hours later. Rhogam (dose is 250ug if <12 weeks for induced or spontaneous abortion) should be administered if the patient is Rh negative. The team should counsel the patient about expected bleeding from medications. Alternative options to medical abortion at the institutional ED include local Planned Parenthood or other clinics that can be found at abortionfinder.org. **Depending on the location of the group performing this simulation, these options for management of an undesired pregnancy may vary and should follow local institutional policy and state regulations**. Throughout, the team maintains a supportive and confidential environment, ensuring that the patient feels safe in discussing her options.

**Critical actions:**

1. Identify the father’s overbearing personality and request a private interview with the patient.
2. Obtain focused history and physical exam with father outside the room, including sexual and obstetric history.
3. Obtain appropriate labs, including STI testing, and imaging for abdominal pain evaluation.
4. Provide appropriate empiric treatment for STIs and ensure follow-up.
5. Maintain a safe, confidential environment for patient disclosure.
6. Senior learners: Provide counseling and management options for undesired pregnancy.

**Case Title:** Human Trafficking presenting as Abdominal Pain

**Chief Complaint:** Patient is a 26-year-old female presenting to the ED for generalized abdominal pain that began five days ago.

**Primary Survey:**

- **Airway:** intact
- **Breathing:** normal, clear bilaterally
- **Circulation:** tachycardic, 2+ symmetric radial pulses bilaterally

**History:**

- **History of present illness:** 26-year-old female presenting to the ED for generalized abdominal pain onset five days. The patient started having severe nausea with some vomiting and has been unable to keep down any food/water over the past 48 hours. The pain is described as constant, dull, and non-radiating. Denies fever, chills, CP, shortness of breath, diarrhea, and vaginal bleeding. She notes some abnormal vaginal discharge in the last week.
- **Past medical history:** G5P1A4 (5 pregnancies, 2 prior medical abortions and 1 surgical abortion, one living 5-year-old child). Multiple sexual partners (male and female). Multiple previous STDs, specifically chlamydia three times in the last year and co-infection with gonorrhea once.
- **Past surgical history:** Surgical abortion.
- **Patient’s medications:** None.
- **Allergies:** No known drug allergy.
- **Social history:** Intravenous heroin use daily since age 19. No EtOH. Sexually active with male and female partners daily since age 17. No barrier or pregnancy protection used. Not currently in college. US citizen.
- **Family history:** father with diabetes

**Secondary Survey/Physical Examination:**

- **General appearance:** fatigue, limited eye contact
- **HEENT:** normal
- **Neck:** supple
- **Heart:** regular and tachycardic, otherwise normal
- **Lungs:** Lungs clear to auscultation bilaterally
- **Abdominal/GI:** mild suprapubic tenderness, no guarding/rebound
- **Genitourinary:** Reported by embedded simulated nurse: os closed, scant gray discharge noted in vaginal vault, positive circular movement tenderness
- **External genitalia:** normal
- **Adnexa:** normal,
- **Uterus:** normal
- **Rectal:** deferred
- **Extremities:** normal
- **Back:** normal
- **Neuro:** normal
- **Skin:** bruising to inner thighs, track marks to bilateral forearms
- **Lymph:** normal
- **Psych:** withdrawn, anxious, constantly asking why things are taking so long

*Image of Left antecubital fossa*

Author’s own image and video

*Image of Left Medial Leg*

Kenny M. Lucie’s bruise after I fell on her. Flickr.

https://www.flickr.com/photos/markkenny/5089482375. Published October 17, 2010.

Accessed September 29, 2025. Original License CC BY-NC-SA 2.0

*Senior Case Ultrasound*

https://www.thepocusatlas.com/ea-obgyn?srsltid=AfmBOopPVU03PAmGlXs8FXSMHv4FZ5yQObSy-c18KhXKAk1ikg7JT_z. Published December 11, 2018. Accessed September 29, 2025. CC BY-NC 4.0

## STANDARDIZED PATIENT BRIEFING MATERIALS

**Case Title:** Human Trafficking presenting as Abdominal Pain

**SP Roles:**

- **Patient: Marisol Morales**
  ○ **Outfit:** For juniors, something to expose arms; for senior case, longer sleeves to prompt a further examination. Initially wear sunglasses in the junior case to be more withdrawn. No sunglasses for the senior case.
- **Father/Trafficker: Mr. Morales** (prefers not to share first name, wants to be addressed as Mr.)
  ○ **Outfit:** Wear something comfortable, maybe a hat or beanie. The goal of clothing is to be as unobtrusive as possible, so it doesn’t want to stand out in the crowd. Would tone down and wear street clothes, be as normal as possible, for example, a button-up shirt and khakis.

**Involvement in the Debrief after the case concludes:**

The Standardized Patient (SP) playing the patient will be in the room after the case; the faculty member that has been in the control booth will enter the sim room with the SP playing the father/trafficker. The faculty will thank both SPs for their assistance with the case.

**Options:**

- If either SP wishes to remain to provide feedback during the debrief at this time, they can join the group by sitting in the two extra chairs provided. Once the initial reactions and discussion on the communication between the learners and you as the SP concludes, the faculty will excuse you with a second thanks and you will exit the room to take a break until the following case.
- If either SP does not wish to stay for the debrief after the thanks, they can immediately exit the room and take a break until the following case.

### Junior R1/R2 Case Considerations

**Presenting History:** 26-year-old female presenting to the emergency department for generalized abdominal pain onset five days. She reports severe nausea with occasional vomiting and has been unable to keep down any food/water over the past 48 hours. The pain is described as constant, dull, and non-radiating. Denies fever, chills, chest pain, shortness of breath, diarrhea, and vaginal bleeding. She notes some abnormal vaginal discharge in the last week. Last menstrual period was three weeks ago.

History is mainly provided by the father, even when questions are directed to the patient. When asked directly, the patient will consistently look to her father for approval before responding to any questions.

The father initially refuses to leave the room for sensitive examination. The physician explains that standard operating procedure allows for a medical chaperone with patient consent if over the age of 18. He will not leave without a team member escorting him out of the room to allow for independent patient evaluation.

**Patient:** After the residents evaluate you, the residents should encourage the father multiple times to leave the room due to need for further examinations, including a pelvic exam. You express concern and alarm, asking “Why shouldn’t my father be here?”

**Exit of the father/trafficker**

3 options:

- residents use the phrase, “we need to do a sensitive exam”
- they ask three times for the father to leave
- or the E(embedded)SP RN moves to get them out

**Tension:** Father/trafficker wants to stay in the room but doesn’t want to make a scene or get others involved.

**Father:** Repeatedly insists that you must remain in the room because your daughter needs your help answering questions. You seem very concerned to the point of nearly yelling, “Why should I leave the room when I’m the only one who knows what is happening here?” Deescalate once the residents explain the need for the sensitive exam and that this is standard procedure to perform on a patient with a medical chaperone if over the age of 18. Continue to insist until a team member escorts you from the room.

FATHER EXITS THE ROOM PRIOR TO THE PHYSICAL EXAM AND COLLECTION OF SOCIAL HISTORY INFORMATION. THIS ALLOWS FOR INDEPENDENT PATIENT EVALUATION AND DISCLOSURE, CRITICAL FOR MEDICAL ASSESSMENT AND SAFETY CONCERNS.

**History/Background:**

- **Name:** Marisol Morales
  **Age:** 26
- **Medical History:** G5P1A4 (five pregnancies, two prior medical abortions and one surgical abortion, one living five-year-old child). Multiple sexual partners (male and female). Multiple previous STDs, specifically chlamydia three times in the last year and co-infection with gonorrhea once.
- **Medications:** None (no birth control use, including barrier protection)
- Allergies: No known drug allergies.
- **Social History:** The patient is a sex worker who reports being coerced by her father, who arranges her clients and controls her earnings. She states that she must earn approximately $2,000 per night to see her five-year-old daughter, with whom her contact is contingent upon meeting this quota. She typically engages with 8–10 clients per night. She reports unprotected sexual activity. The patient discloses ongoing heroin use, administered via injection, and notes that her father provides her heroin. She has abstained from use for the past five days due to illness. She describes using heroin to cope with the trauma of her experiences and expresses a desire to quit but feels unable to do so because of its ready availability and lack of support. The patient reports feeling unsafe at home and expresses fear that disclosure of her situation could result in harm to herself or her daughter. She denies current suicidal or homicidal ideation but reports persistent depression and anxiety related to her living situation and ability to see her daughter.
- **Family History:** Father with diabetes

**Physical Exam:**

- **Vitals:** BP 124/68 HR 109 RR 20 Pulse ox 98% Temp 98.8º F
- **General:** appears fatigued and withdrawn with limited eye contact, wearing sunglasses
- **HEENT:** normal
- **Neck:** supple
- **Lung:** normal
- **Card:** normal
- **Abd:** mild suprapubic tenderness, no guarding/rebound, tolerates palpation
- **Neuro:** alert, oriented, and appropriate
- **Skin:** bruising to inner thighs (says “oh I bruise easily”), track marks to bilateral arms
- **GU:** os closed, scant gray discharge noted in vaginal vault, negative cervical motion tenderness (CMT), reported by embedded simulated nurse, no exam performed on SP
- **Psych:** withdrawn, anxious, constantly asking why things are taking so long

**Patient:** The pelvic exam will show that you have vaginal discharge. Cry during the discussion of the exam findings and exclaim, “Do I have a disease? That would impact my life and my job.” Residents will probe further, and they will provide support/encouragement. As you feel more comfortable, you can provide some history regarding what your job is.

**Patient:** Disclosure of your full social history:

Wait until the resident specifically asks for and builds trust and rapport with you.

Second option when you are upset about the discharge/infection and how it will affect your job for junior case.

Once reassured, express that you do not feel safe at home. You are not currently suicidal, but you have constant anxiety and depression related to when you will see your daughter next and concerns for your safety.

Residents will explain to the patient the need for empiric treatment for sexually transmitted infections and expanded testing including Gonorrhea/Chlamydia/Trichomonas/HSV/Syphilis/HIV.

**Patient:** Agree to HIV testing and empiric treatment.

Residents should provide a safe environment for the patient and ensure confidentiality. Discuss options with the patient, including a call to the national human trafficking hotline (NHTH) or referral to local county law enforcement or social work.

**Patient:** Decline involvement of social work, the National Human Trafficking Hotline, or Law Enforcement at this time. You may state you do not feel safe to leave your father at this time and are worried that extended time without him in the room could result in punishment or losing access to your daughter. Thank the physician for treatment and request discharge. You should be offered strategies to access the contact numbers later, and you agree to memorize them so that you can safely reach out when it is safe for you and your daughter.

### Senior R3/R4 Case Considerations

**Presenting History:** 26-year-old female presenting to the emergency department for generalized abdominal pain onset five days. The patient started having severe nausea with some vomiting and has been unable to keep down any food/water over the past 48 hours. The pain is described as constant, dull, and non-radiating. Denies fever, chills, chest pain, shortness of breath, diarrhea, and vaginal bleeding. She notes some abnormal vaginal discharge in the last week. Missed her last menstrual period.

History is mainly provided by the father, even when questions are directed to the patient. When asked directly, the patient will consistently look to her father for approval before responding to any questions.

The father initially refuses to leave the room for sensitive examination. The physician explains that standard operating procedure allows for a medical chaperone with patient consent if over the age of 18. He will not leave without a team member escorting him out of the room to allow for independent patient evaluation.

**Patient:** After the residents evaluate you, the residents should encourage the father multiple times to leave the room due to need for further examinations, including a pelvic exam. You express concern and alarm, asking “Why shouldn’t my father be here?”

**Exit of the father/trafficker**

3 options:

- residents use the phrase “we need to do a sensitive exam”
- they ask three times for the father to leave
- or the ESP RN moves to get them out

**Tension:** Father/trafficker wants to stay in the room but doesn’t want to make a scene or get others involved

**Father:** Repeatedly insist that you must remain in the room because your daughter needs your help answering questions. You seem very concerned to the point of nearly yelling, “Why should I leave the room when I’m the only one who knows what is happening here?” Deescalate once the residents explain the need for the sensitive exam and that this is standard procedure to perform on a patient with a medical chaperone if over the age of 18. Continue to insist until a team member escorts you from the room.

FATHER EXITS THE ROOM PRIOR TO THE PHYSICAL EXAM AND COLLECTION OF SOCIAL HISTORY INFORMATION. THIS ALLOWS FOR INDEPENDENT PATIENT EVALUATION AND DISCLOSURE, CRITICAL FOR MEDICAL ASSESSMENT AND SAFETY CONCERNS.

**History/Background:**

- **Name:** Marisol Morales
  **Age:** 26
- **Medical History:** G5P1A4 (five pregnancies, two prior medical abortions and one surgical abortion, one living five-year-old child). Multiple sexual partners (male and female). Multiple previous STDs, specifically chlamydia three times in the last year and co-infection with gonorrhea once.
- **Medications:** None (no birth control use, including barrier protection)
- **Allergies:** NKDA (no known drug allergies)
- **Social History:** The patient is a sex worker who reports being coerced by her father, who arranges her clients and controls her earnings. She states that she must earn approximately $2,000 per night to see her five-year-old daughter, with whom her contact is contingent upon meeting this quota. She typically engages with 8–10 clients per night. She reports unprotected sexual activity, which has likely resulted in her current pregnancy. The patient discloses ongoing heroin use, administered via injection, and notes that her father provides her heroin. She has abstained from use for the past five days due to illness. She describes using heroin to cope with the trauma of her experiences and expresses a desire to quit but feels unable to do so because of its ready availability and lack of support. The patient reports feeling unsafe at home and expresses fear that disclosure of her situation could result in harm to herself or her daughter. She denies current suicidal or homicidal ideation but reports persistent depression and anxiety related to her living situation and ability to see her daughter.
- Family History: Father with diabetes

**Physical Exam:**

- **Vitals:** BP 124/68 HR 109 RR 20 Pulse ox 98% Temp 98.8º F
- **General:** appears fatigued and withdrawn with limited eye contact
- **HEENT:** normal
- **Neck:** supple
- **Lung:** normal
- **Card:** normal
- **Abd:** mild suprapubic tenderness, no guarding/rebound, tolerates palpation
- **Neuro:** alert, oriented, and appropriate
- **Skin:** bruising to inner thighs (says “oh I bruise easily”), track marks to bilateral arms
- **GU:** os closed, scant gray discharge noted in vaginal vault, negative CMT (reported by embedded simulated nurse, no exam performed on SP)
- **Psych:** withdrawn, anxious, constantly asking why things are taking so long

**Patient:** The pelvic exam will show that you have vaginal discharge. Cry during the discussion of the exam findings and exclaim, “Do I have a disease? That would impact my life and my job.” Residents will probe further, and they will provide support/encouragement.

**Patient:** Disclosure of your full social history:

Wait until the resident specifically asks for and builds trust and rapport with you

Second option when you are upset about the discharge/infection and how it will affect your job for junior case.

Third option in senior case when you learn about pregnancy.

As you feel more comfortable, you can provide some history regarding what your job is. Once reassured, express that you do not feel safe at home. You are not currently suicidal, but you have constant anxiety and depression related to when you will see your daughter next and concerns for your safety.

Residents will explain to the patient the need for empiric treatment for sexually transmitted infections and expanded testing including Gonorrhea/Chlamydia/Trichomonas/HSV/Syphilis/HIV.

**Patient:** Agree to HIV testing and empiric treatment.

Residents will also inform you that your pregnancy test is positive.

**Patient:** Begin crying again and stating: “I can’t be pregnant. I can’t bring another child into my life. Please help me.” You request an abortion. (This aspect of the case may need to be altered due to the local state regulations of the institution implementing the case.)

Patient response to counseling for Senior case with undesired pregnancy consideration: If they don’t give you a good reason to tell your father/trafficker why you need to follow up with OB, you can suggest the idea that it is for the infection rather than pregnancy. If they don’t counsel you that this medication for the abortion will cause heavy vaginal bleeding, you can say you remember bleeding the last time and ask if that will happen again.

Residents will explain the institutional protocol, noting that a transvaginal ultrasound (TVUS) is needed. If TVUS shows an intrauterine pregnancy (IUP) of less than ten weeks and after checking your Rh status, a medical abortion can be pursued. The residents then will need to consult an obstetrician to see you in the emergency department to give the first medication.

**Patient:** You express your wish to terminate again and agree to follow up with OB. You state that you will just tell your father the appointment is for the infection found today. You make it clear that you do not want your father to know you are pregnant. You ask if the OB visit can be framed as treatment for the infection to prevent your father from finding out. You also ask if you should be worried about bleeding, referencing heavy bleeding during a previous pregnancy.

Residents provide a safe environment for patients and ensure confidentiality. Discuss options with the patient, including a call to the national human trafficking hotline (NHTH) or referral to local county law enforcement or social work. Place the referral to OB.

**Patient:** Decline involvement of social work, the National Human Trafficking Hotline, or Law Enforcement at this time. You may state you do not feel safe to leave your father at this time and are worried that extended time without him in the room could result in punishment or losing access to your daughter. Thank the physician for treatment and request discharge. You will be offered strategies to access the contact numbers later, and you agree to memorize them so that you can safely reach out when it is safe for you and your daughter.

## OPERATOR MATERIALS

**Diagnosis:**

Pelvic Inflammatory Disease (PID)

**Disposition:**

Home

## DEBRIEFING AND EVALUATION PEARLS

### Human Trafficking presenting as Abdominal Pain

**Pearls:** The simulation case on human trafficking in emergency medicine provides valuable learning points for residents, helping them develop essential skills and knowledge to recognize and respond to trafficking situations effectively. The debriefing session is intentionally structured to reinforce these pearls, allowing learners to reflect on their performance, connect to evidence-based frameworks, and consolidate new skills. Here are some key learning points:

1. **Recognizing Red Flags**: Residents learn to identify potential indicators of human trafficking, such as unexplained injuries, inconsistent stories, signs of physical or psychological trauma, and accompanying individuals exhibiting controlling behavior. These red flags were emphasized in the case and revisited during debriefing with examples drawn from the simulation encounter.
2. **Trauma-Informed Care**: Residents understand the importance of providing trauma-informed care (TIC) to trafficking survivors, emphasizing empathy, non-judgmental communication, and sensitivity to the unique needs and experiences of victims. TIC is a relatively new paradigm in healthcare. According to the Substance Abuse and Mental Health Services Administration (SAMHSA), the six guiding principles are: safety, trustworthiness and transparency, peer support, collaboration and mutuality, empowerment and choice, and sensitivity to cultural, historical, and gender issues.^13^ In this simulation, learners are prompted to practice TIC by asking permission before sensitive questions or exams, maintaining a calm and supportive tone, giving patients control over disclosure, and creating a safe environment. During debriefing, facilitators explicitly connect these behaviors back to the TIC framework, reinforcing both theoretical foundations and clinical application.
3. **Patient-Centered Approach**: Residents practice engaging with patients in a patient-centered manner, prioritizing trust-building, active listening, and empowerment while respecting patient autonomy and confidentiality. Facilitators highlight that patient-centered care in trafficking cases requires balancing medical management with the patient’s readiness to disclose or accept resources.
4. **Navigating Ethical Dilemmas**: Residents navigate ethical dilemmas related to patient confidentiality, mandatory reporting requirements, and the balance between preserving patient autonomy and ensuring victim safety. Debriefing focuses on applying ethical principles to realistic scenarios, with attention to local laws, institutional policies, and cultural considerations.^14^
5. **Effective Communication Skills**: Residents hone their communication skills, including asking open-ended questions, validating patient concerns, and providing clear explanations of available resources and support options. Special emphasis is placed on nonjudgmental language and the avoidance of retraumatizing phrases, consistent with TIC.
6. **Documentation and Reporting**: Residents understand the importance of accurate and detailed documentation of findings related to potential trafficking cases, including documenting physical injuries, behavioral observations, disclosures made by patients, and creating a confidential chart. Faculty stress that documentation should be objective, precise, and carefully worded because these records may later serve legal or protective purposes.
7. **Resources Available**: Residents recognize the National Human Trafficking Hotline (phone number: 1-888-373-7888, text: 233733) as a resource when they identify a victim of human trafficking. Available 24/7, the hotline offers confidential support and guidance in multiple languages to survivors, concerned community members, and professionals. Healthcare providers can contact the hotline to report suspected trafficking cases, seek consultation on appropriate responses, and access referrals to local service providers and law enforcement agencies. In the debrief, participants are encouraged to role-play how they would discreetly share this information with patients while prioritizing safety.

**Other debriefing points:** Debriefing following a human trafficking simulation is a pivotal opportunity for participants to engage in reflective dialogue, share insights, and identify areas for growth. An experienced debriefer is essential for guiding learners through the clinical, ethical, and emotional complexities of this case. If new faculty or simulationists are running the case, consultation with institutional resources such as social work, psychiatry, or chaplaincy is strongly recommended. Participants are encouraged to acknowledge and discuss any emotional responses evoked during the simulation, validating feelings of discomfort or uncertainty. Faculty may normalize these reactions as part of working with vulnerable populations while also reinforcing professional strategies for managing emotional burden.

Observations and insights from the encounter are explored, focusing on the identification of red flags and indicators of human trafficking in the healthcare setting. Communication strategies, such as active listening and empathetic engagement, are reviewed alongside discussions on decision-making processes, interdisciplinary collaboration, and adherence to institutional protocols. Participants are prompted to reflect on their individual strengths and areas for improvement, setting personalized learning goals for future development.

Resources and support services available for healthcare providers addressing human trafficking are also reviewed, with emphasis on self-care and advocacy. Facilitators encourage learners to consider not only the immediate patient interaction but also their role as advocates for systemic improvements within emergency medicine. The debriefing session concludes with a commitment to applying learned skills and knowledge to real-world practice, advocating for systemic changes, and supporting trafficking survivors within their communities. Facilitated in a supportive environment, the debriefing fosters collaborative learning and reinforces participants’ dedication to addressing this critical issue in emergency medicine.

## SIMULATION ASSESSMENT

*Human Trafficking presenting as Abdominal Pain*

Learner: _________________________________________

***Assessment Timeline***

This timeline is to help observers assess their learners. It allows observer to make notes on when learners performed various tasks, which can help guide debriefing discussion.

**Critical Actions:**

□ Identify the father’s overbearing personality and request a private interview with the patient.
□ Obtain focused history and physical exam with father outside the room, including sexual and obstetric history.
□ Obtain appropriate labs, including STI testing and imaging for abdominal pain evaluation.
□ Provide appropriate empiric treatment for STIs and ensure follow-up.
□ Maintain a safe, confidential environment for patient disclosure.

**Summative and formative comments:**

**Milestones assessment:**
