# Supplementary material for: Abdominal Pain and Vaginal Discharge: An Eye-Opening Simulation Case about Human Trafficking
Source: J Educ Teach Emerg Med. 2025 Oct 31;10(4):S1–S41. doi: 10.21980/J8.52150 (PMC12594474; doi:10.21980/J8.52150)
Supplement: Supplementary file 1 [file 10-4-S1-Supp1.pptx]

## Slide 1
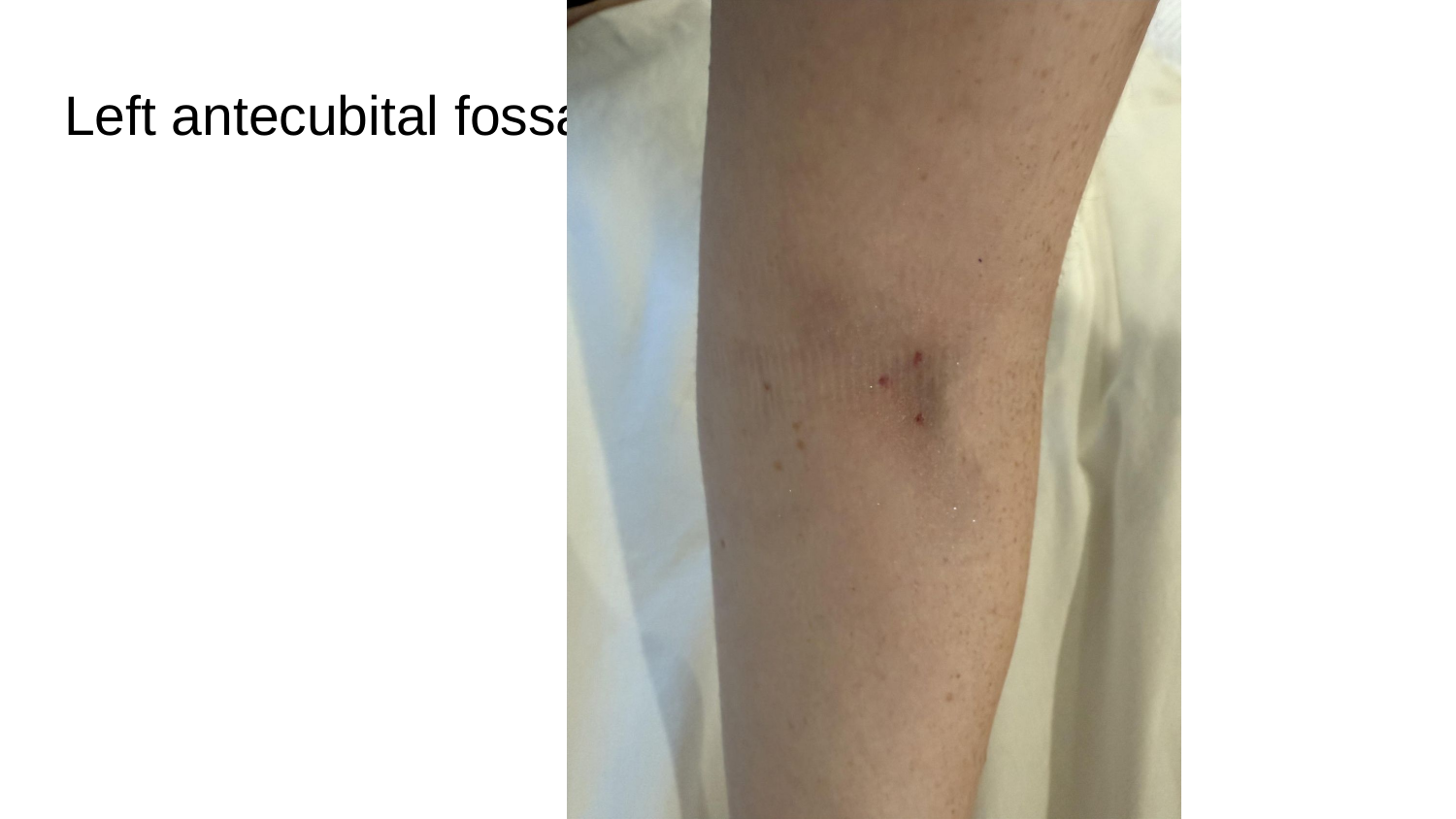

# Left antecubital fossa

## Slide 2
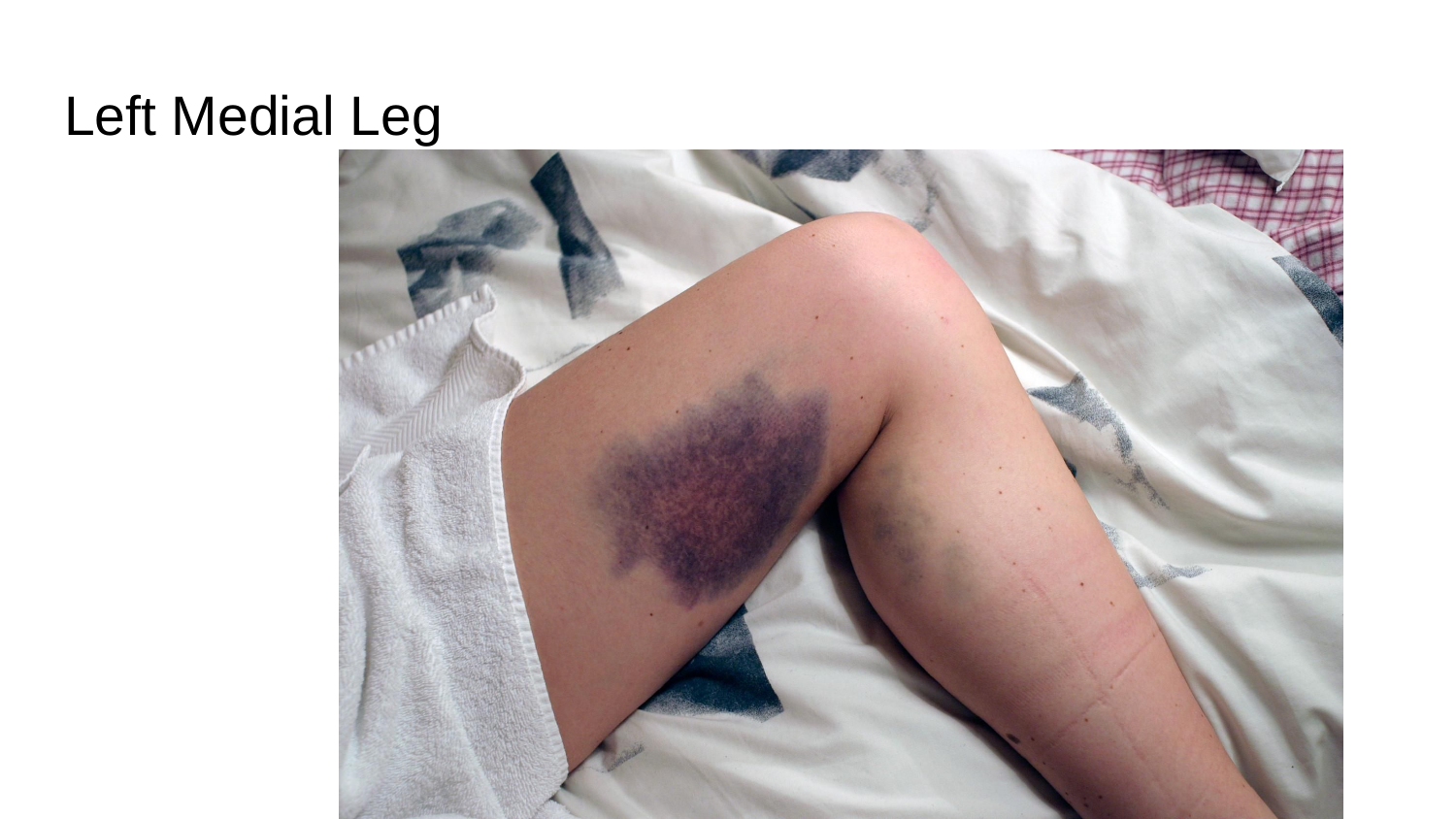

# Left Medial Leg

## Slide 3
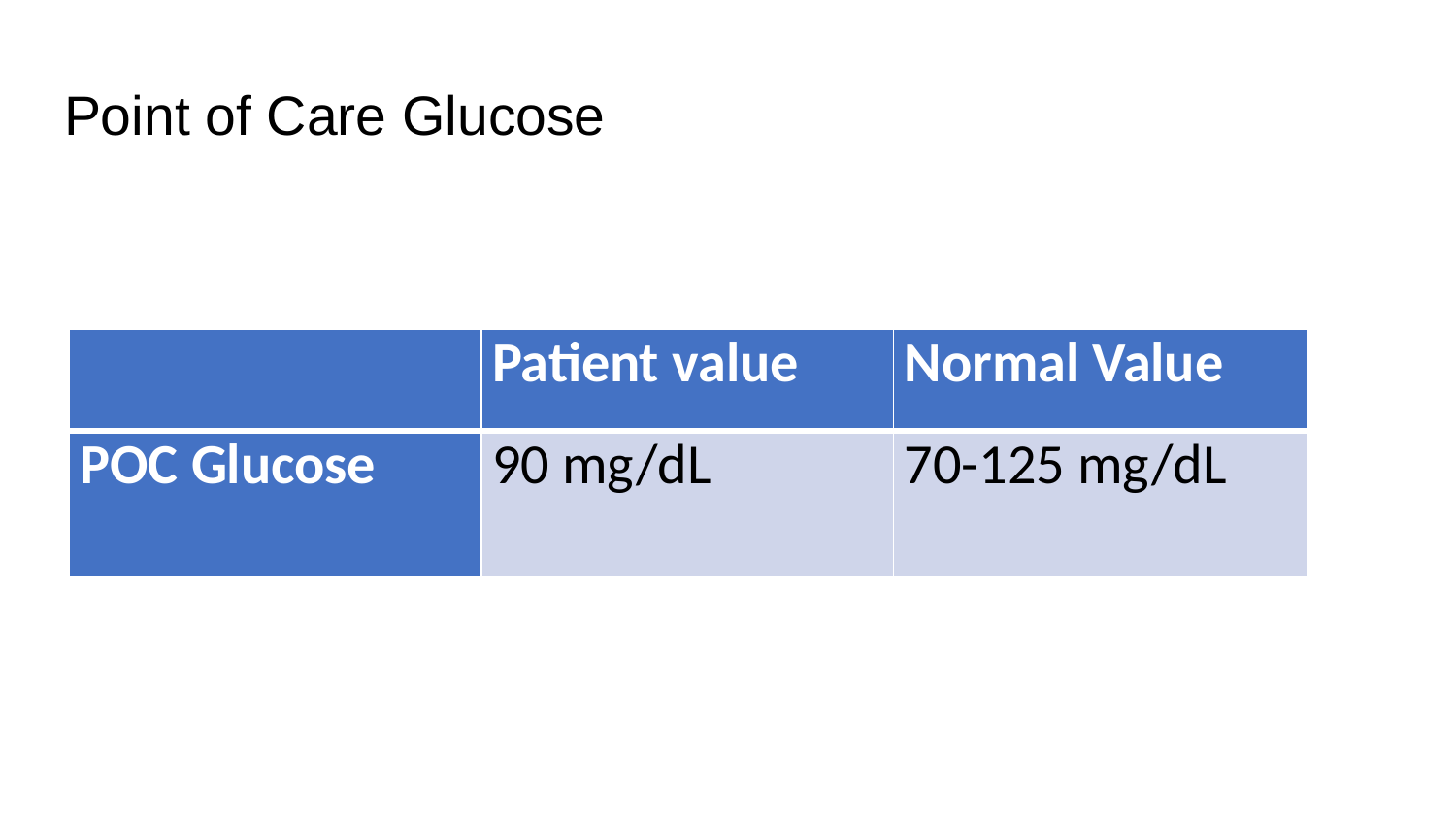

# Point of Care Glucose
| | Patient value | Normal Value |
| --- | --- | --- |
| POC Glucose | 90 mg/dL | 70-125 mg/dL |

## Slide 4
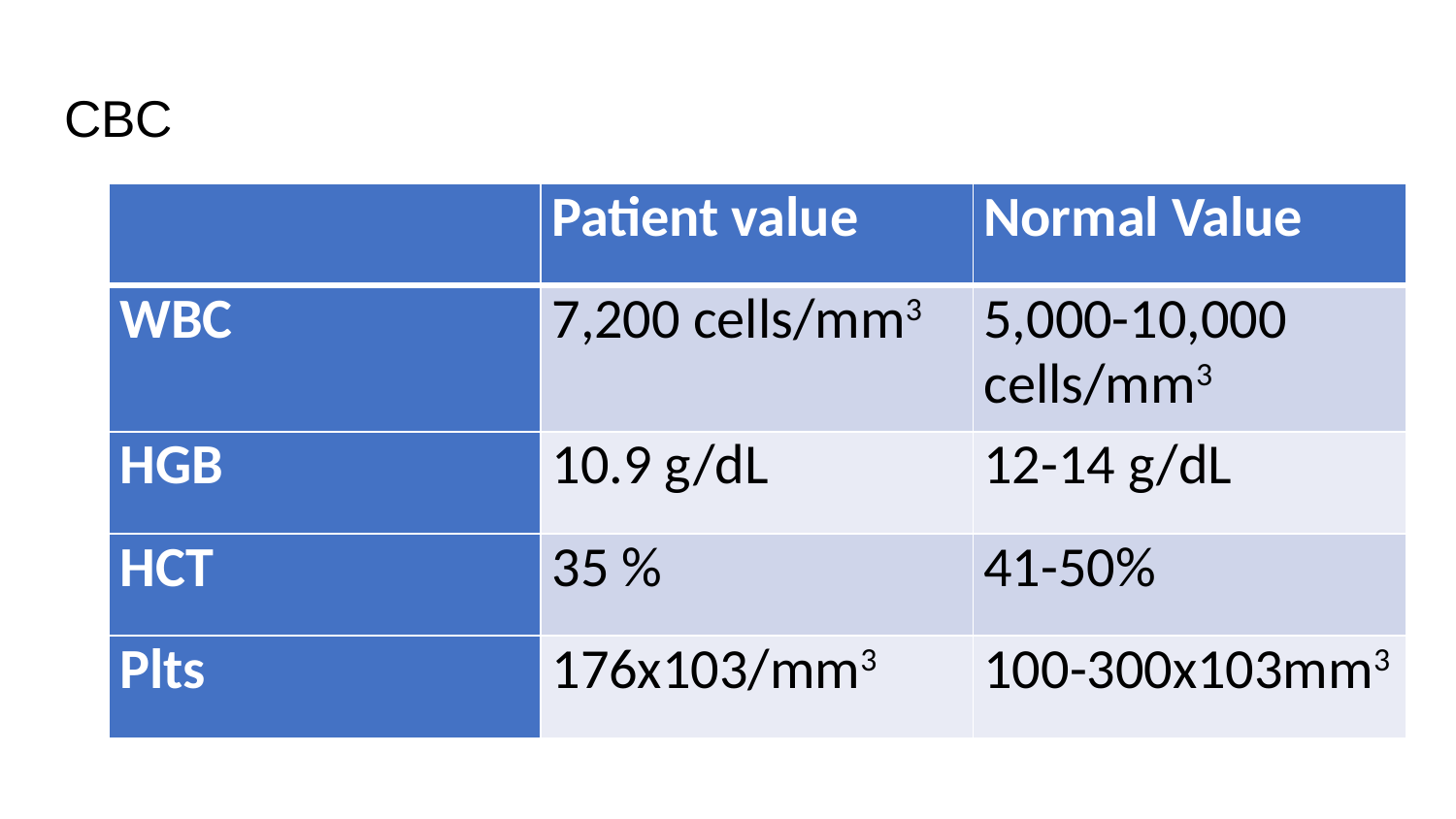

# CBC
| | Patient value | Normal Value |
| --- | --- | --- |
| WBC | 7,200 cells/mm3 | 5,000-10,000 cells/mm3 |
| HGB | 10.9 g/dL | 12-14 g/dL |
| HCT | 35 % | 41-50% |
| Plts | 176x103/mm3 | 100-300x103mm3 |

## Slide 5
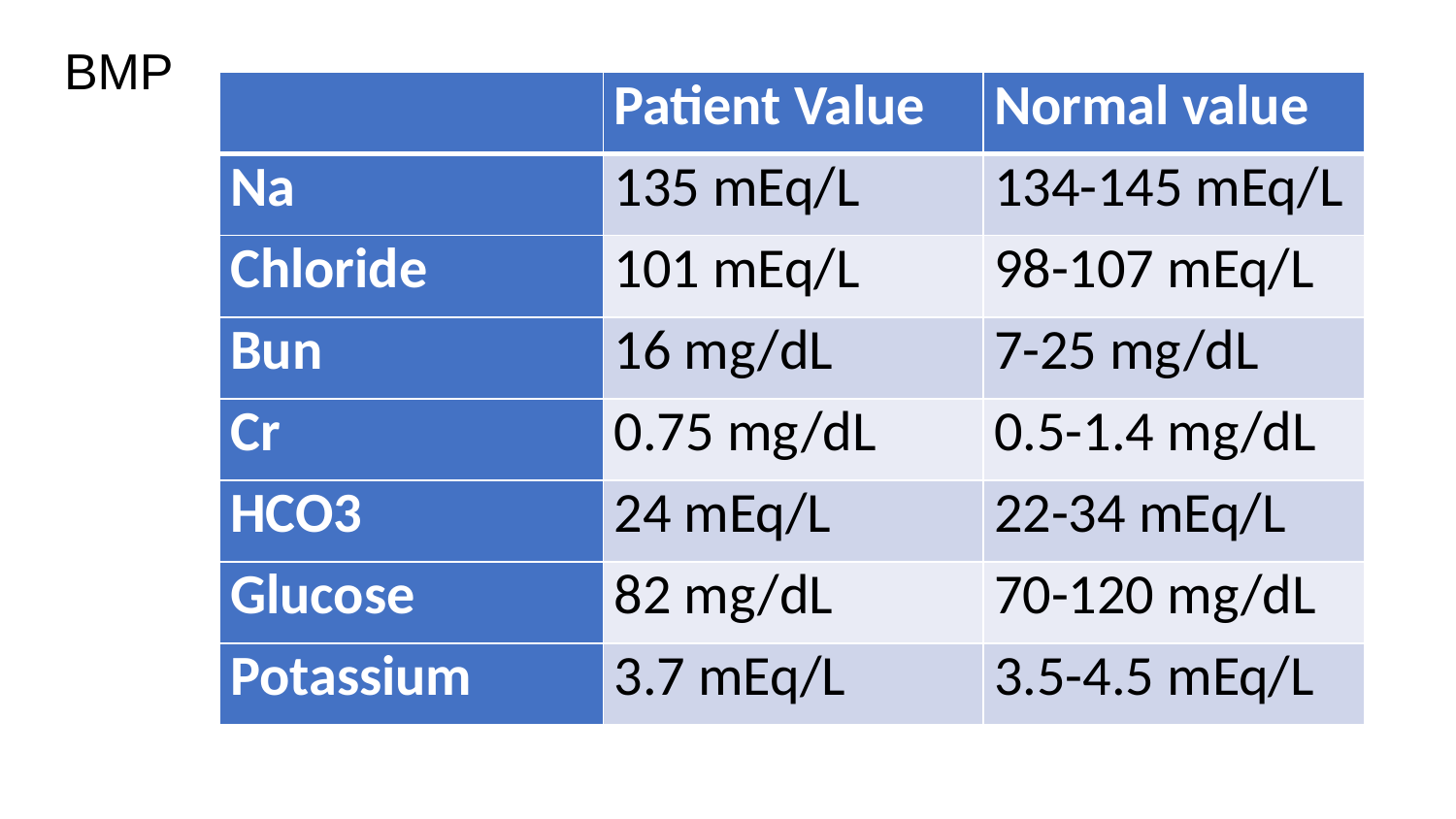

# BMP
| | Patient Value | Normal value |
| --- | --- | --- |
| Na | 135 mEq/L | 134-145 mEq/L |
| Chloride | 101 mEq/L | 98-107 mEq/L |
| Bun | 16 mg/dL | 7-25 mg/dL |
| Cr | 0.75 mg/dL | 0.5-1.4 mg/dL |
| HCO3 | 24 mEq/L | 22-34 mEq/L |
| Glucose | 82 mg/dL | 70-120 mg/dL |
| Potassium | 3.7 mEq/L | 3.5-4.5 mEq/L |

## Slide 6
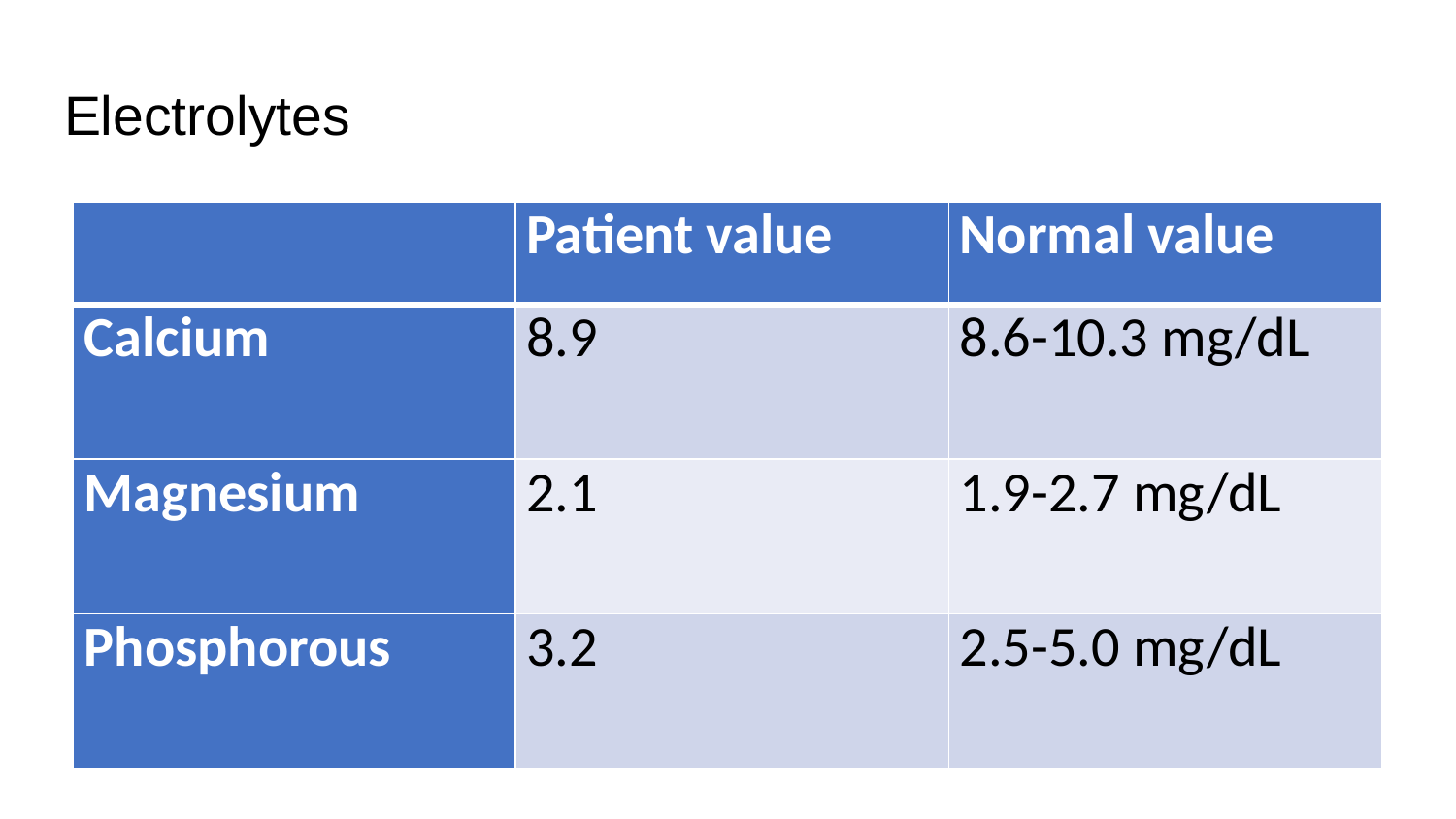

# Electrolytes
| | Patient value | Normal value |
| --- | --- | --- |
| Calcium | 8.9 | 8.6-10.3 mg/dL |
| Magnesium | 2.1 | 1.9-2.7 mg/dL |
| Phosphorous | 3.2 | 2.5-5.0 mg/dL |

## Slide 7
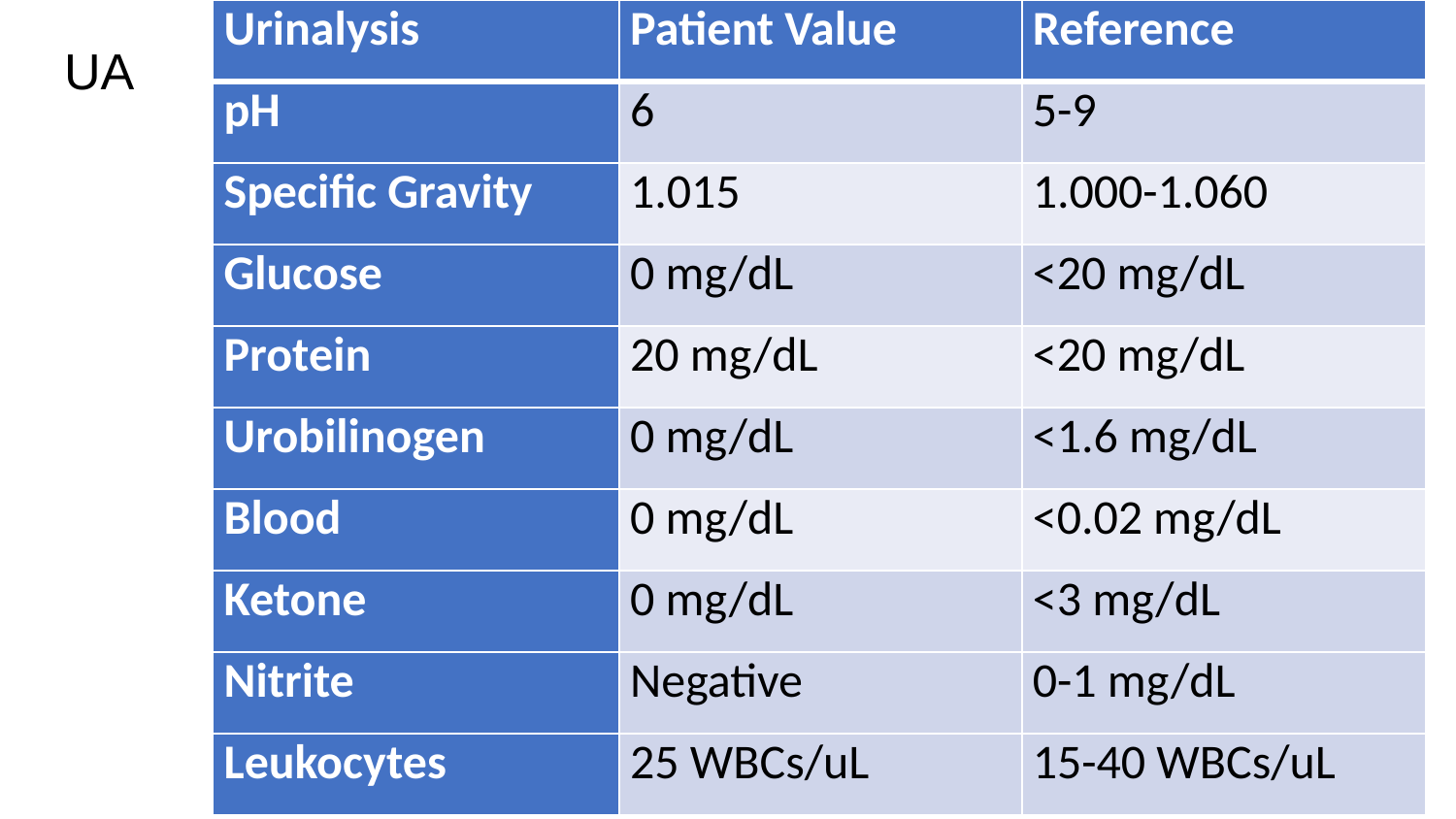

| Urinalysis | Patient Value | Reference |
| --- | --- | --- |
| pH | 6 | 5-9 |
| Specific Gravity | 1.015 | 1.000-1.060 |
| Glucose | 0 mg/dL | <20 mg/dL |
| Protein | 20 mg/dL | <20 mg/dL |
| Urobilinogen | 0 mg/dL | <1.6 mg/dL |
| Blood | 0 mg/dL | <0.02 mg/dL |
| Ketone | 0 mg/dL | <3 mg/dL |
| Nitrite | Negative | 0-1 mg/dL |
| Leukocytes | 25 WBCs/uL | 15-40 WBCs/uL |
# UA

## Slide 8
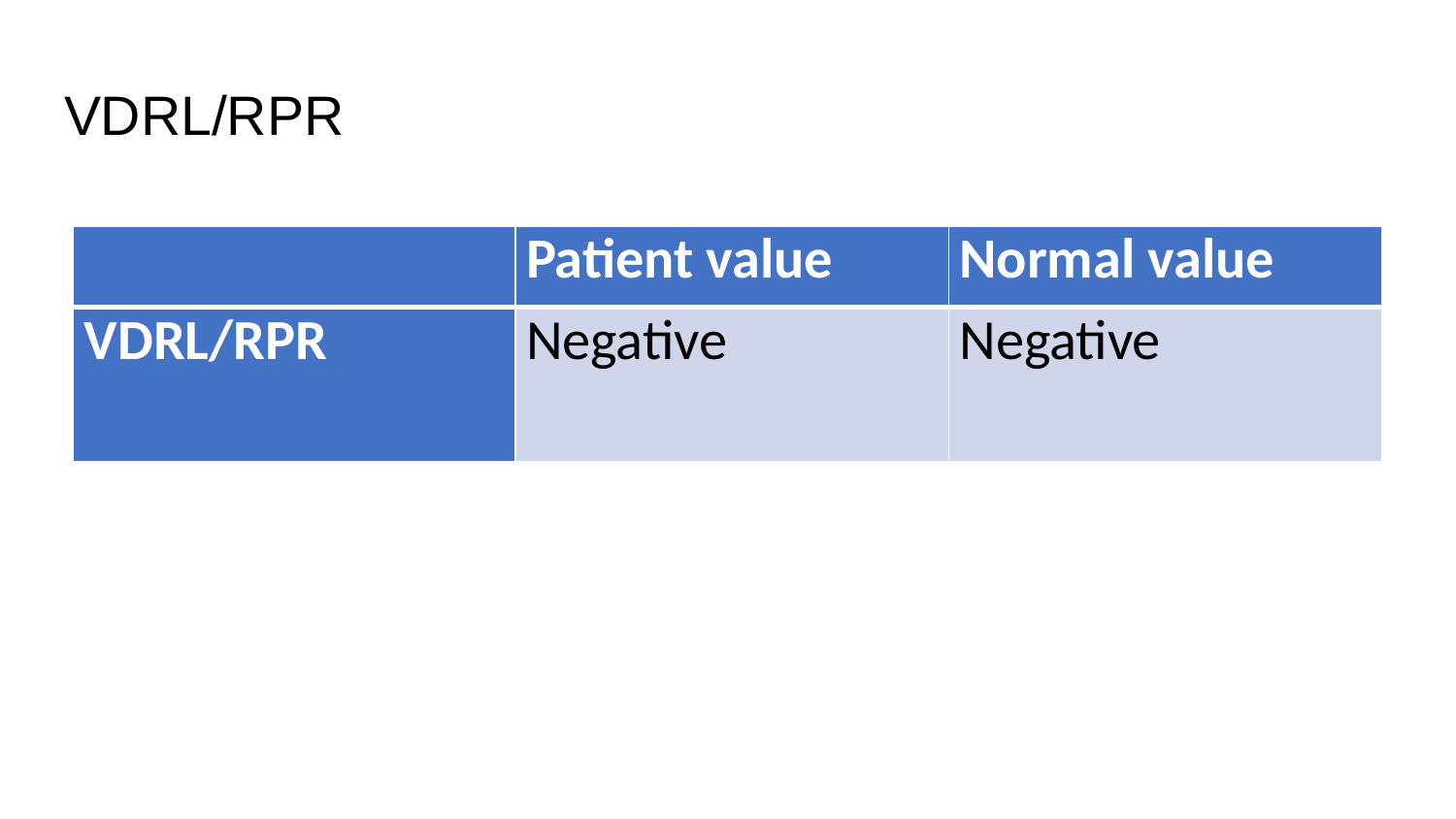

# VDRL/RPR
| | Patient value | Normal value |
| --- | --- | --- |
| VDRL/RPR | Negative | Negative |

## Slide 9
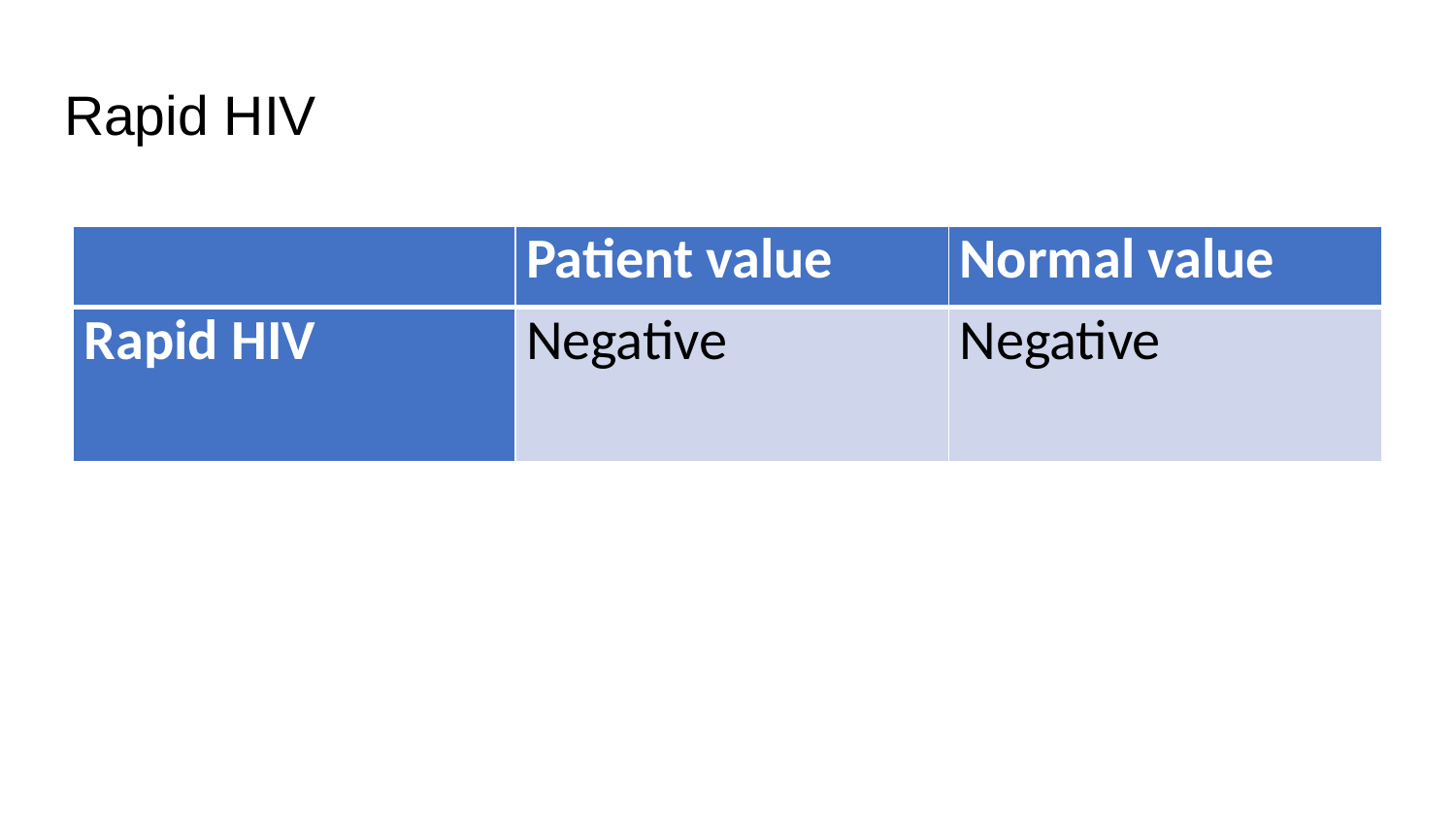

# Rapid HIV
| | Patient value | Normal value |
| --- | --- | --- |
| Rapid HIV | Negative | Negative |

## Slide 10
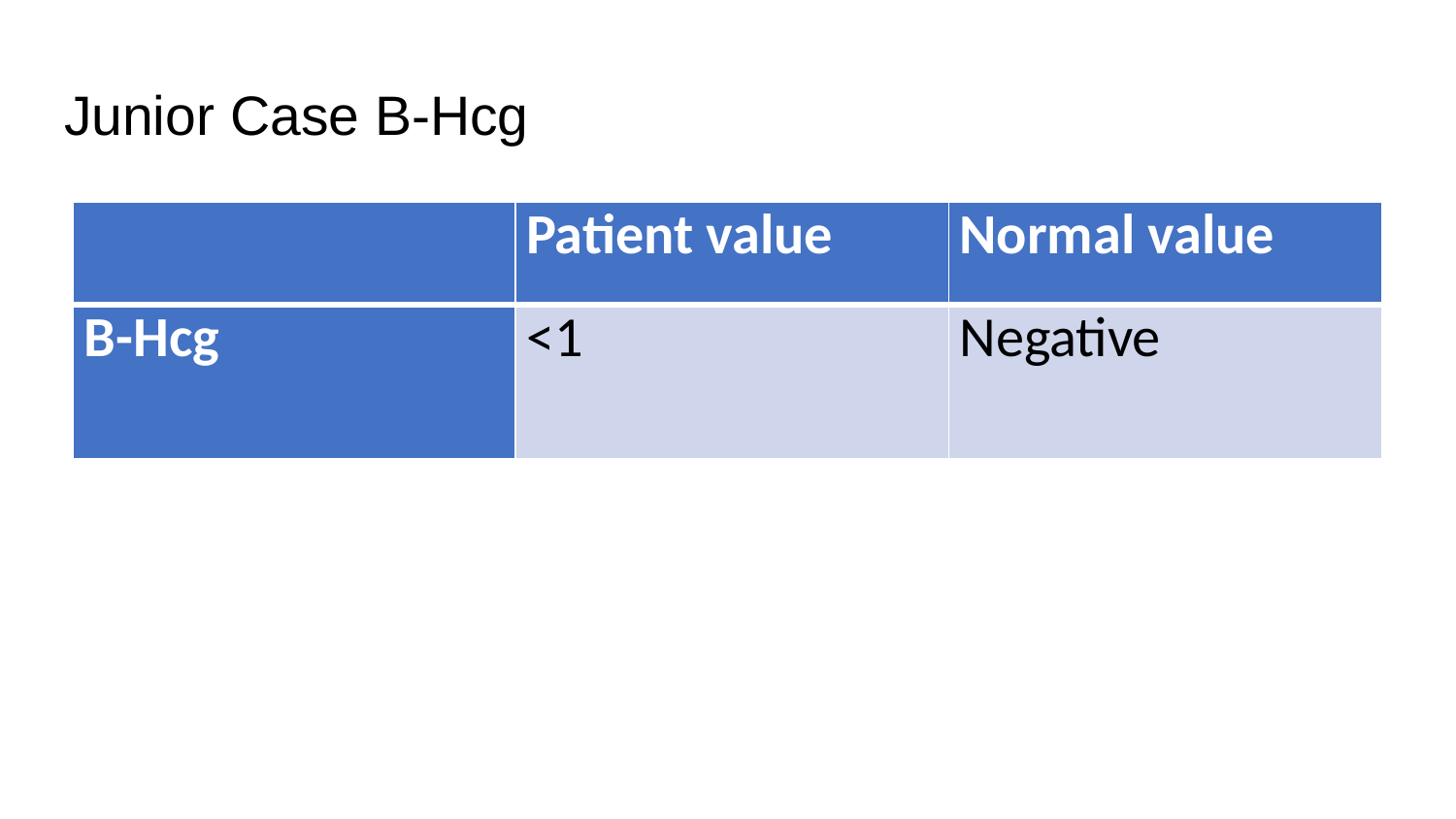

# Junior Case B-Hcg
| | Patient value | Normal value |
| --- | --- | --- |
| B-Hcg | <1 | Negative |

## Slide 11
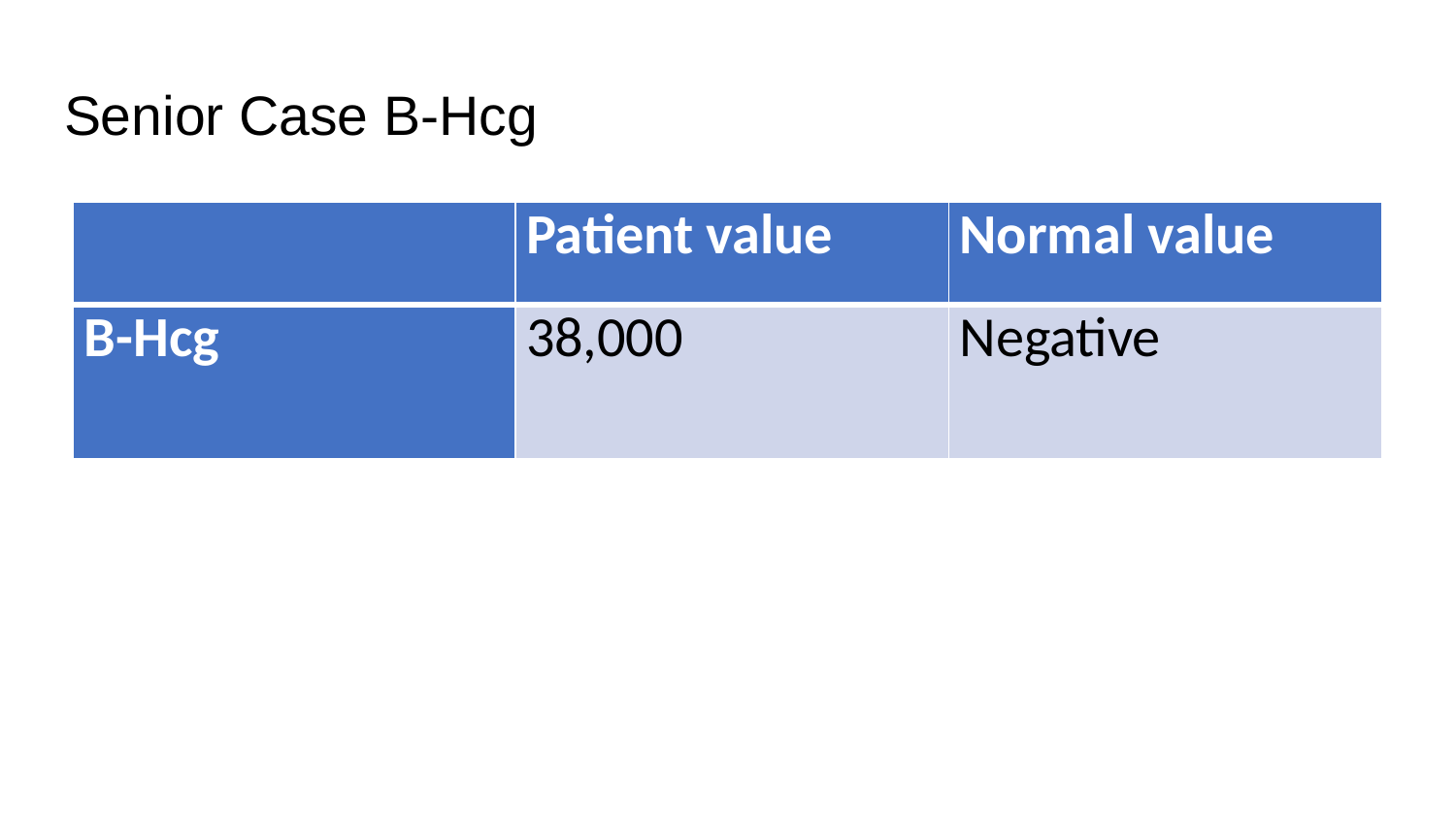

# Senior Case B-Hcg
| | Patient value | Normal value |
| --- | --- | --- |
| B-Hcg | 38,000 | Negative |

## Slide 12
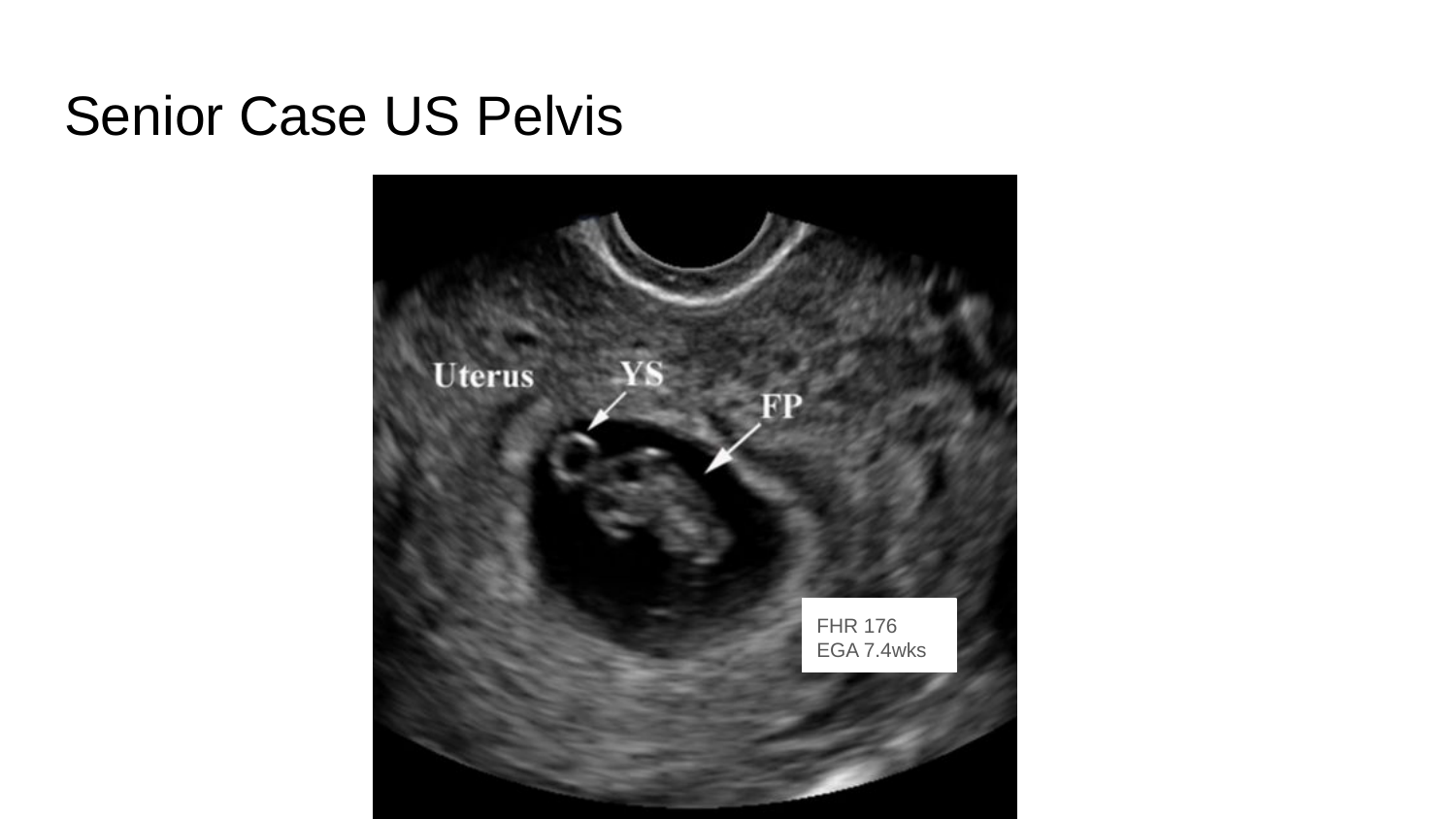

# Senior Case US Pelvis
FHR 176
EGA 7.4wks

## Slide 13
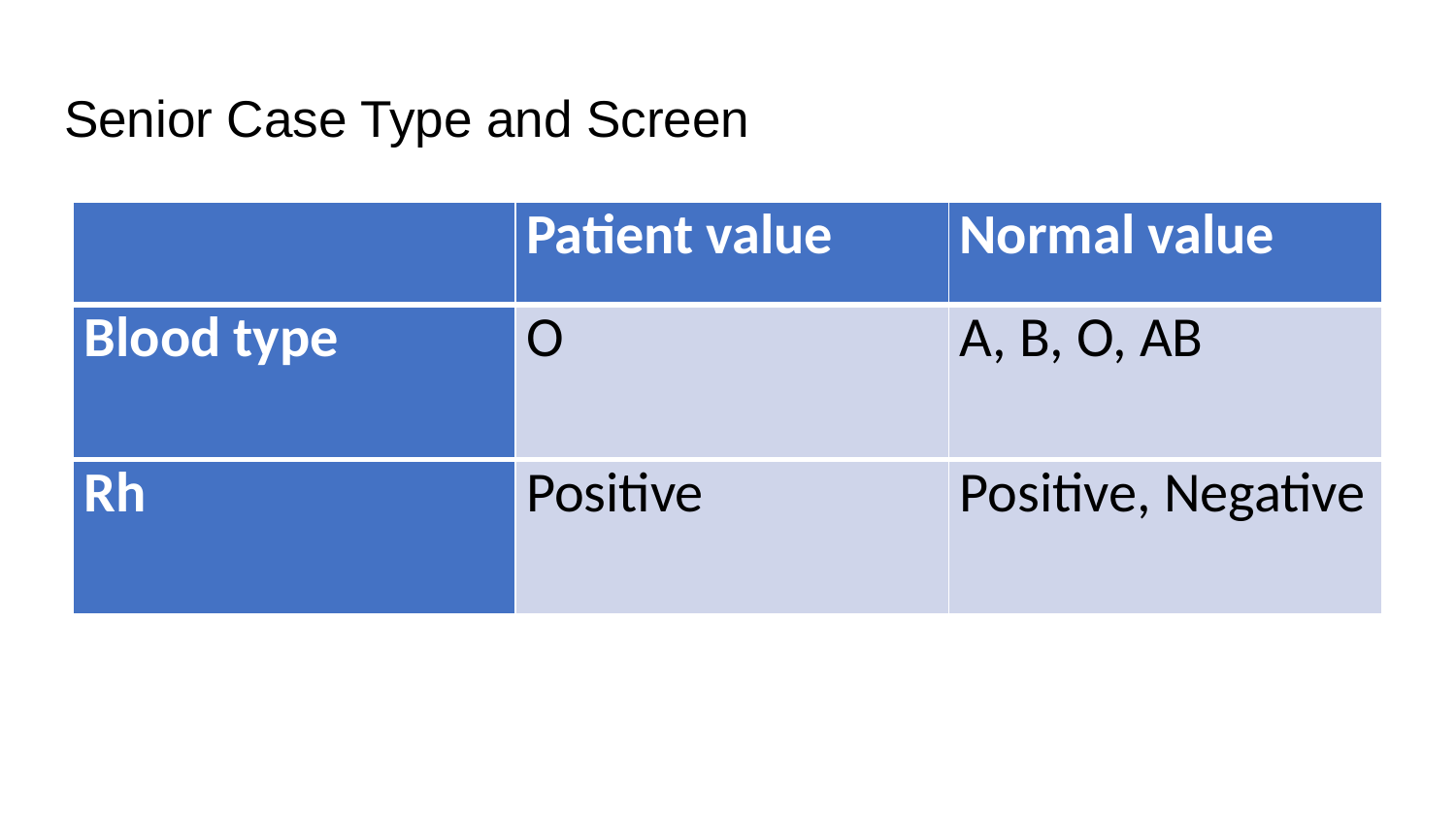

# Senior Case Type and Screen
| | Patient value | Normal value |
| --- | --- | --- |
| Blood type | O | A, B, O, AB |
| Rh | Positive | Positive, Negative |
